# NIK promotes metabolic adaptation of glioblastoma cells to bioenergetic stress

**DOI:** 10.1038/s41419-020-03383-z

**Published:** 2021-03-15

**Authors:** Michael L. Kamradt, Ji-Ung Jung, Kathryn M. Pflug, Dong W. Lee, Victor Fanniel, Raquel Sitcheran

**Affiliations:** 1 Department of Molecular & Cellular Medicine, Texas A&M University Health Science Center, College Station, TX 77845 USA; 2 Medical Sciences Graduate Program, Texas A&M University Health Science Center, College Station, TX 77845 USA; 3 Interdisciplinary Graduate Program in Genetics, Texas A&M University, College Station, TX 77845 USA; 4 Present Address: Department of Pharmacology, University of Texas Southwestern Medical Center, Dallas, TX 75390 USA

**Keywords:** Cancer metabolism, Energy metabolism, Cancer metabolism

## Abstract

Cancers, including glioblastoma multiforme (GBM), undergo coordinated reprogramming of metabolic pathways that control glycolysis and oxidative phosphorylation (OXPHOS) to promote tumor growth in diverse tumor microenvironments. Adaptation to limited nutrient availability in the microenvironment is associated with remodeling of mitochondrial morphology and bioenergetic capacity. We recently demonstrated that NF-κB-inducing kinase (NIK) regulates mitochondrial morphology to promote GBM cell invasion. Here, we show that NIK is recruited to the outer membrane of dividing mitochondria with the master fission regulator, Dynamin-related protein1 (DRP1). Moreover, glucose deprivation-mediated metabolic shift to OXPHOS increases fission and mitochondrial localization of both NIK and DRP1. NIK deficiency results in decreased mitochondrial respiration, ATP production, and spare respiratory capacity (SRC), a critical measure of mitochondrial fitness. Although IκB kinase α and β (IKKα/β) and NIK are required for OXPHOS in high glucose media, only NIK is required to increase SRC under glucose deprivation. Consistent with an IKK-independent role for NIK in regulating metabolism, we show that NIK phosphorylates DRP1-S616 in vitro and in vivo. Notably, a constitutively active DRP1-S616E mutant rescues oxidative metabolism, invasiveness, and tumorigenic potential in NIK^−/−^ cells without inducing IKK. Thus, we establish that NIK is critical for bioenergetic stress responses to promote GBM cell pathogenesis independently of IKK. Our data suggest that targeting NIK may be used to exploit metabolic vulnerabilities and improve therapeutic strategies for GBM.

## Introduction

Cancer cells adapt to survive and grow in continuously changing, and often harsh, tumor microenvironments characterized by acidity, hypoxia, and limited availability of nutrients, such as amino acids and glucose. Indeed, metabolic adaptability of aggressive cancers to these diverse growth conditions is increasingly recognized as a major factor contributing to therapy resistance. While cancer cells generally prefer to use aerobic glycolysis to support growth, many cancers, including glioblastoma multiforme (GBM), can meet their bioenergetics demands utilizing both glycolysis and oxidative mitochondrial metabolism^1,2^. Mitochondria are highly dynamic signaling organelles that are integral for sensing and adapting to changes in the cellular microenvironment for optimal growth and survival, providing energy in the form of adenosine triphosphate (ATP) produced by oxidative phosphorylation (OXPHOS)^3,4^. A well-recognized bioenergetic parameter of mitochondrial fitness is the spare respiratory capacity (SRC), which is the difference between the maximum and basal mitochondrial oxygen consumption rates (OCRs). A robust SRC allows cells to accommodate changes in energy demands such as increased cell proliferation, as well as respond to stresses in the tumor microenvironment, such as nutrient deprivation^5,6^.

Continuous remodeling of mitochondrial morphology allows cells to respond rapidly to metabolic cues and is achieved by a dynamic balance between mitochondrial fission and fusion^7,8^. Mitochondrial fission requires dynamin-related protein 1 (DRP1), which is predominantly cytosolic, but is recruited to the outer mitochondrial membrane (OMM), where it oligomerizes, constricts, and fissions larger mitochondria into smaller ones^9^. Recruitment of DRP1 to mitochondria is a highly regulated process that involves its phosphorylation by kinases that respond to several growth and stress signals, with phosphorylation at serine 616 (pDRP1-S616) being critical for its fission-promoting activity^10–12^. Notably, mitochondrial fission is often induced in response to cellular stress^13,14^ and a highly fragmented mitochondrial network is observed in many cancer cells^15,16^. Importantly, preventing mitochondrial fission through DRP1 knockdown impairs cancer cell growth, and increased DRP1 expression promotes migration and invasion of multiple cancer types^15–17^. Therefore, regulation of DRP1 activity and mitochondrial fission are critical processes in cancer cells.

NF-κB-inducing kinase (NIK; *MAP3K14*) is best known for its roles in immunity and inflammation^18^. It has become increasingly clear that NIK has a wide range of functions in human diseases ranging from bone and autoimmune disorders to cancer^19^. We have previously discovered that a discrete pool of NIK, a key upstream regulator of noncanonical NF-κB signaling, colocalizes to mitochondria in GBM, as well as other cancer cells^20^. Furthermore, we demonstrated that NIK regulates DRP1 mitochondrial localization and promotes mitochondrial fission, motility, and subcellular trafficking to enhance GBM cell migration and invasion^20^. However, a role for NIK in regulating mitochondrial metabolism and metabolic reprogramming has not been investigated. Here, we show that NIK regulates GBM mitochondrial dynamics and metabolism in response to bioenergetic stress resulting from glucose deprivation and substitution of galactose as a carbon source, which inhibits glycolysis and forces dependence on mitochondrial OXPHOS^21^. Our results demonstrate that NIK is a key regulator of DRP1 and is required for metabolic reprogramming to increase mitochondrial OCR and SRC and promote GBM tumorigenesis.

## Results

### NIK is an outer membrane mitochondrial protein that co-localizes with DRP1 at mitochondrial fission sites in vivo

**Fig. 1:**
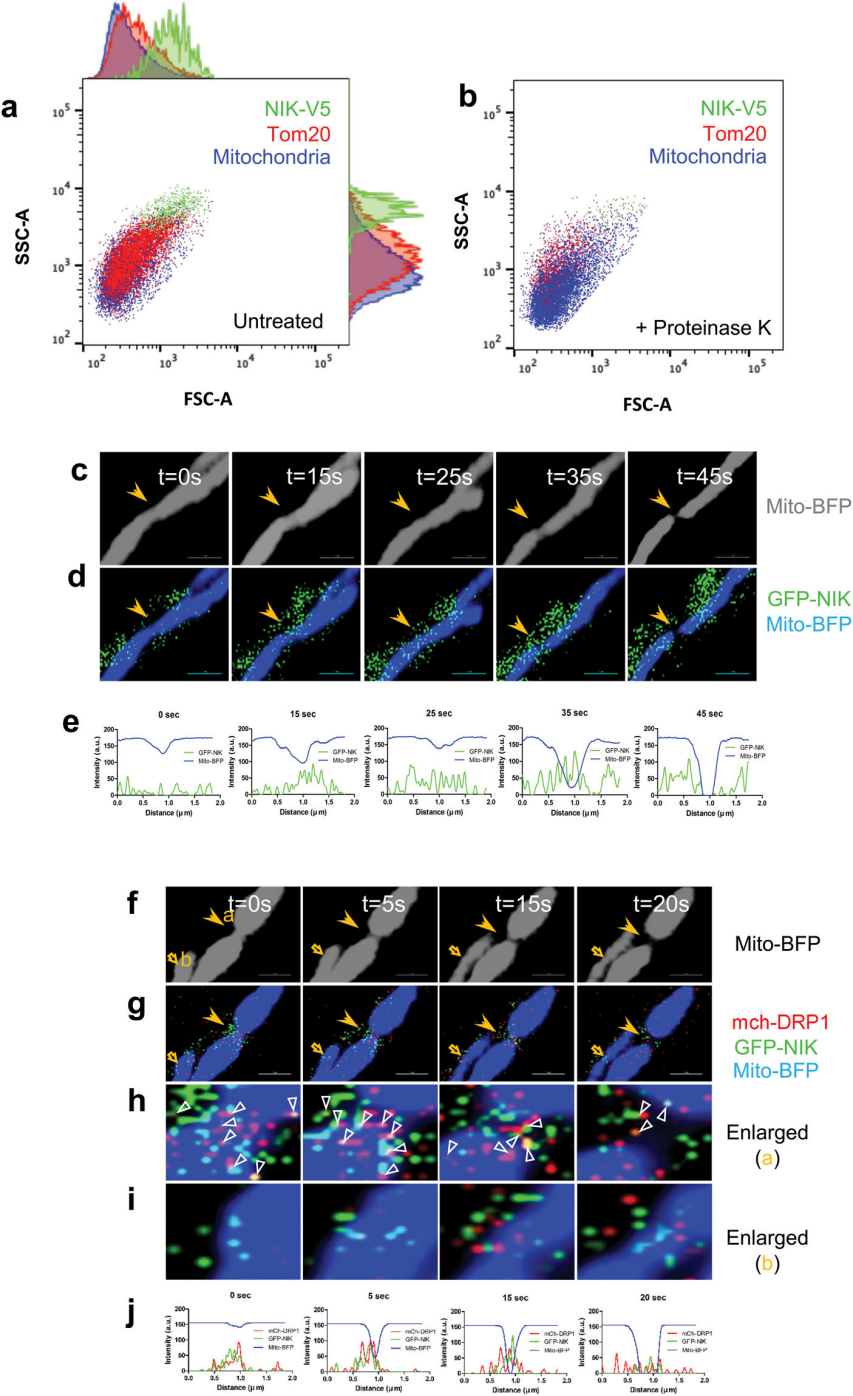
NIK is a mitochondrial outer membrane protein that co-localizes with DRP1 at mitochondrial fission sites in vivo. **a**,**b** Representative protease protection assay was performed using single organelle flow cytometry analysis of untreated (**a**) and Proteinase K-treated mitochondria (**b**) isolated from BT25 cells expressing V5-tagged NIK (NIK-V5) and stained with AF647-conjugated V5 antibody (pseudocolored green), Tom20-AF488 conjugated antibody (pseudocolored red) and MitoSOX™ (pseudocolored blue). NIK-V5-, Tom20-, and MitoSOX™-positive organelles were analyzed by forward scatter (FSC-A) and side scatter (SSC-A) to evaluate mitochondrial size and complexity. See Supplementary Fig. 1 for bead calibration and standard curve calibration of flow cytometer parameters to calculate mitochondrial size. **c**–**e** Time-lapse images of BT25 cell transiently expressing mito-BFP and GFP-NIK were acquired at 5 s intervals for 3 min. **c** Grayscale images show mito-BFP fluorescence with arrow indicating fission site. **d** GFP-NIK signal (green) is shown merged with mito-BFP (blue). Scale bar, 2 μm. **e** Line-scan analysis of mean fluorescence intensity shows highest GFP-NIK localization at the mitochondrial fission site (lowest mito-BFP signal). **f**–**j** Time-lapse image of BT25 cells transiently expressing mito-BFP, mch-DRP1, and GFP-NIK acquired at 5 s intervals for 3 min. **f** Grayscale images show mito-BFP fluorescence with filled arrow indicating fission site and open arrow indicating a constriction site. **g** mch-DRP1 (red) and GFP-NIK (green) are shown merged with mito-BFP (blue). Scale bar, 2 μm. **h** Enlargement of the mitochondrial fission site indicated by the closed arrow (a) in **f** shows co-localization of mch-DRP1 with GFP-NIK. Open arrowheads indicate colocalization of mch-DRP1 and GFP-NIK. **i** Enlargement of the mitochondrial constriction site indicated by the open arrow (b) in **f** shows NIK (green) and DRP1 (red) recruitment to mitochondria. **j** Line-scan analysis of mean fluorescence intensity shows highest GFP-NIK and mch-DRP1 localization at the mitochondrial fission site (lowest mito-BFP signal). Images were threshold-adjusted by subtracting background GFP or mcherry signal in the cytosol, followed by deconvolution. The images shown are a representative of live cell imaging of mitochondria (*n* > 5), respectively. See also Fig. S2.

We previously reported that a pool of NIK protein localizes to mitochondria and is required for maximal mitochondrial recruitment of DRP1^20^. To further characterize the mitochondrial localization of NIK, we performed single organelle flow cytometry analysis. Mitochondrial-enriched fractions were isolated from BT25 GBM cells expressing V5-tagged NIK (NIK-V5) and labeled with MitoSox™ Red, a superoxide specific dye that accumulates in the mitochondrial matrix. Fractions were either left untreated or treated with Proteinase K to degrade proteins on the outer surface of intact mitochondria. Flow cytometry analysis revealed that a substantial fraction of mitochondria (pseudocolored blue) were NIK-V5 positive (green) (Fig. 1a). However, in contrast with the mitochondrial resident protein Tom20 (red), which colocalized with almost all mitochondria (Fig. 1a), NIK-V5 was predominantly associated with larger, more complex mitochondria that have increased forward and side scatter (Fig. 1a and Supplementary Fig. 1a, b). Notably, similar to Tom20, NIK-V5 signal was significantly reduced after digestion of OMM proteins with Proteinase K (Fig. 1b). These findings were further supported by the results of a protease protection immunoblot assay on mitochondrial preparations obtained from NIK-V5-expressing GBM cells (Supplementary Fig. 1b, c). Together, these results suggest that a pool of NIK protein localizes to the outer membrane of large mitochondria, consistent with a role for NIK in regulating mitochondrial fission.

To evaluate the subcellular localization of NIK in vivo, we performed live imaging of GBM cells expressing a green fluorescent protein fused to NIK (GFP-NIK). GFP-NIK is fully competent to activate the non-canonical NF-κB pathway, including phosphorylation of IKKα/β, p100 processing to p52, and p52 nuclear translocation (Supplementary Fig. 1d). Time-lapse confocal fluorescence microscopy of Mito-BFP-labeled mitochondria undergoing fission (Fig. 1c, d) revealed that GFP-NIK accumulates transiently at mitochondrial constriction sites, and then re-distributes across the mitochondria shortly after fission (Fig. 1d, arrows). Fluorescence intensity plots show that GFP-NIK signal increases during fission and is highest at the site of mitochondrial constriction where mitochondrial Mito-BFP signal is lowest (Fig. 1e). Since DRP1 accumulates at mitochondrial constriction sites immediately prior to fission, we assessed the subcellular localization of mCherry-tagged DRP1 (mch-DRP1) together with GFP-NIK during early mitochondrial constriction (Fig. 1f, open arrow) and after fission was completed (Fig. 1f, closed arrow). Time-lapse images revealed that NIK and DRP1 co-localize at the outer surface of constriction (Fig. 1g, open arrows) and fission sites (Fig. 1g, closed arrows, and Fig. 1h, open arrowheads). Colocalization of mch-DRP1 with GFP-NIK was observed at the outer surface of the constriction sites (closed arrows, Fig. 1g and open arrowheads, Fig. 1h). GFP-NIK and DRP1 also accumulate at future mitochondrial constriction sites (see open arrows and enlarged inset in Fig. 1h, i), suggesting that these events are necessary for fission. Fluorescence intensity plots verified a strong co-localization between GFP-NIK and mch-DRP1, with highest levels at sites of fission where Mito-BFP signal is lowest (Fig. 1j). Although the correlation of DRP1 and NIK signals at mitochondrial fission sites is strong, only a small number of yellow-orange punctate signals was observed and many green and red signals were adjacent, but not overlapping, possibly indicative of transient interactions. Recruitment of NIK and DRP1 into mitochondrial fission sites was also observed in COS-7 cells (Supplementary Fig. 2a, b), demonstrating that the co-localization of these proteins is not limited to GBM cells. Consistent with NIK-DRP1 co-localization, immunoprecipitation assays demonstrated that both ectopic and endogenously expressed NIK form a complex with DRP1 (Supplementary Fig. 3a, b). These results suggest that NIK enrichment at mitochondrial constriction sites plays an important role in initiating mitochondrial fission through its interaction with, and recruitment of DRP1.

### Metabolic shift from glycolysis to OXPHOS triggers NIK-dependent mitochondrial fission

**Fig. 2:**
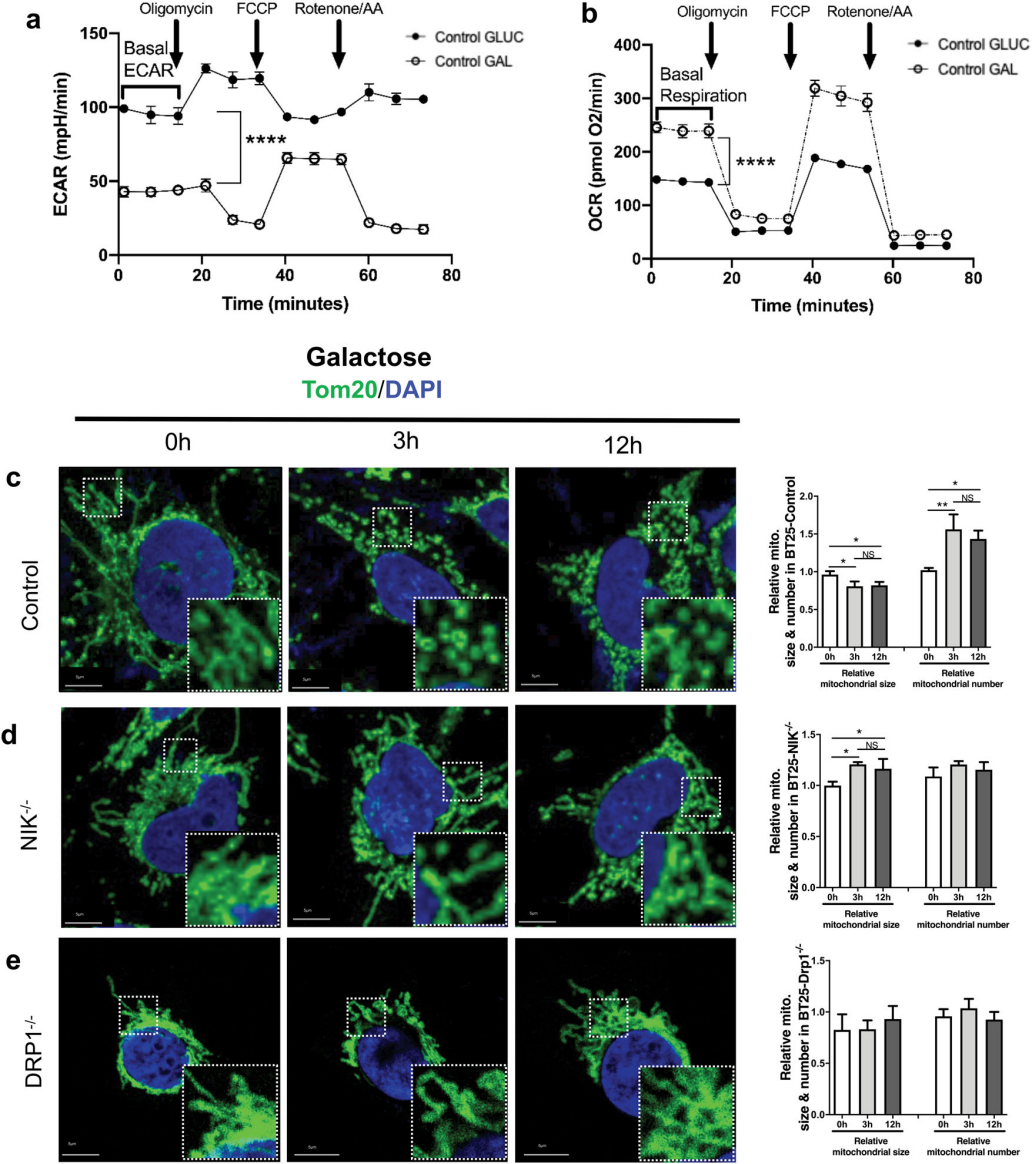
Metabolic shift from glycolysis to OXPHOS triggers NIK-dependent mitochondrial fission. **a** Representative Seahorse XF Cell Mito Stress test showing extracellular acidification rate (ECAR) of BT25 control cell in glucose and galactose media. Bracket indicates significance between basal ECAR between the conditions. Student’s *t* test (paired, two tailed) *p* < 0.0001. **b** Change in O_2_ consumption rate (OCR) plotted as pmol O_2_ per minute. Brackets indicate significantly different basal respiration. Student’s *t* test (paired, two-tailed) *p* < 0.0001. **c**–**e** Representative confocal images of BT25 control, NIK^−/−^, and DRP1^−/−^ cells grown in 18 mM glucose and shifted to 18 mM galactose for the indicated time points and immunostained with a Tom20-FITC-conjugated antibody. Areas marked with white squares are amplified and shown in the insert. Scale bar, 5 μm. DRP1 knockdown was verified by immunoblot (Supplementary Fig. 4) and NIK^−/−^ cells were described previously^20^. Quantification of mitochondria size and number of control, NIK^−/−^, and DRP1^−/−^ cells pictured to the right of each image respectively. Mitochondria was quantified using Image J and significance was determined by one-way ANOVA followed by Tukey post-hoc analysis (*n* ≥ 30 cells per cell condition); *p < 0.05, **p < 0.01, ns: not significant.

Mitochondrial morphology is highly dynamic and is influenced by metabolic stimuli^22–24^. To explore whether NIK plays a role in regulating changes in mitochondrial morphology and metabolism in response to different bioenergetic demands, we used Seahorse XF analyzer MitoStress assays to evaluate mitochondrial function in GBM cells cultured under different nutrient conditions to induce a metabolic shift from glycolysis to forced reliance on OXPHOS^21,25^. When cells were glucose starved and shifted to galactose as a carbon source, they exhibited decreased extracellular acidification rates (ECAR), indicative of reduced glycolysis (Fig. 2a). Moreover, GBM cells efficiently adapted to glucose deprivation and growth in galactose by increasing OCR (Fig. 2b). Confocal immunofluorescence microscopy revealed that mitochondria in control cells undergo fragmentation in galactose growth conditions after 3 h, persisting up to 12 h (Fig. 2c). However, galactose-induced mitochondrial fragmentation was impaired in NIK^−/−^ and DRP1^−/−^ GBM (Fig. 2d, e, respectively), which was reflected in quantification of mitochondria size and number. These results demonstrate that glucose deprivation and substitution with galactose induces a metabolic shift in GBM cells to OXPHOS and triggers mitochondrial fission that is dependent on NIK and DRP1.

### Forced reliance on OXPHOS increases mitochondrial accumulation of NIK

**Fig. 3:**
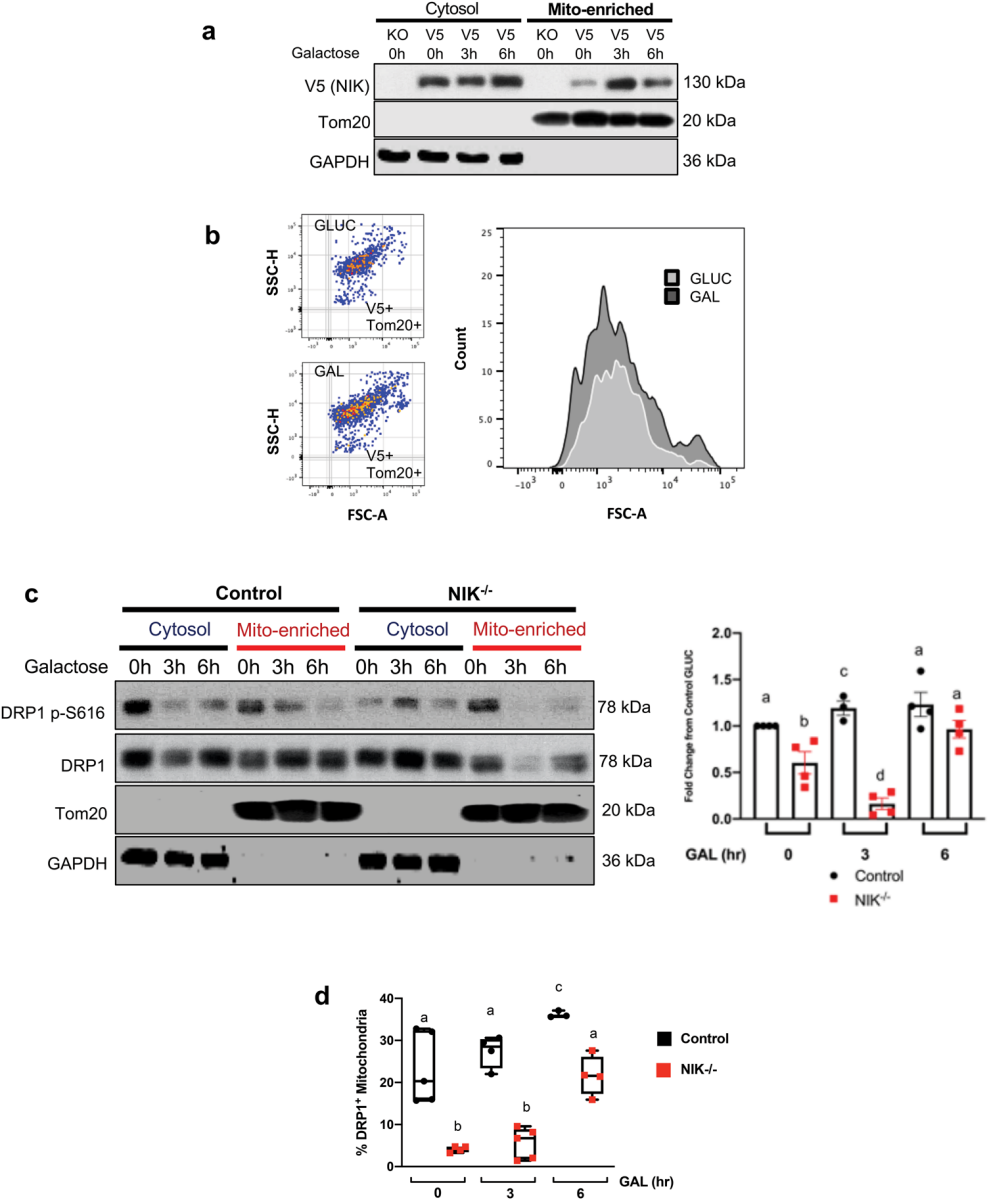
Forced reliance on OXPHOS increases mitochondrial accumulation of NIK. **a** Representative immunoblot analysis of cytosolic and mitochondrial subcellular fractions in BT25 NIK^−/−^ cells reconstituted with NIK conjugated to V5 tag (NIK-V5) after metabolic switch from glucose to galactose media was performed with indicated antibodies. **b** Representative single organelle flow cytometry analysis was performed on BT25 NIK-V5 cells cultured in 18 mM glucose (top left) or 18 mM galactose (bottom left) and immunostaining with mitochondrial Tom20 (AF488) and V5 (AF647). (Right) histogram demonstrating the increase in NIK-V5+ mitochondria upon switch to 18 mM galactose media for 6 h. Individual mitochondria analyzed for glucose conditions *n* = 20,268 and individual mitochondria for galactose conditions, *n* = 20,242. **c** Immunoblot analysis was performed with indicated antibodies using cytosolic and mitochondrial subcellular fractions from BT25 control and NIK^−/−^ cells shifted from glucose to galactose at the indicated times (left). Quantification of *n* = 3–4 independent biological replicates of total DRP1 western blot by densitometry analysis in Image J shown in **c**. Individual Student’s *t* tests were performed between conditions. Different letters indicate statistically significant differences; “a vs b”, “a vs c”, “b vs c”, and “b vs d” *p* < 0.05; “a vs d” *p* < 0.001 and “c vs d” *p* < 0.0001. **d** Single organelle flow cytometry analysis of DRP1-AF488 recruitment at MitoSox™ Red^+^ mitochondria. Each cell/condition was compared to DRP1^−/−^ as a negative control to determine the level of mitochondria enrichment. Individual Student’s *t* tests were performed between conditions. Different letters indicate statistically significant differences; “a vs b” and “b vs c” *p* < 0.0001, “a vs c” *p* < 0.01.

Strikingly, we observed that forcing GBM cells to rely on mitochondrial metabolism by switching from glucose to galactose media increases accumulation of NIK at mitochondria within 3 h (Fig. 3a). Single organelle analysis of mitochondria confirmed that NIK-V5 mitochondrial signal increased by ~2-fold in galactose-cultured GBM cells (Fig. 3b). Additionally, DRP1 protein levels decreased in the cytosol and increased in mitochondria at 3 h after switching the cells to galactose (Fig. 3c). However, DRP1 mitochondrial localization was decreased in GBM cells lacking NIK (NIK^−/−^) in glucose (0 h galactose timepoint, Fig. 3d) and DRP1 mitochondrial localization further diminished after switching to galactose medium (Fig. 3d). We also found that NIK^−/−^ had decreased DRP1 serine 616 phosphorylation (pDRP1-S616), which has previously been shown to be critical for mitochondrial fission. Single organelle analysis confirmed that while the percent of DRP1-positive mitochondria increased with galactose-induced OXPHOS in control cells, the overall levels and the kinetics of DRP1 mitochondrial localization were impaired in NIK^−/−^ cells (Fig. 3d). Loss of mitochondrial DRP1 is consistent with the more fused mitochondria that we observed in NIK^−/−^ cells (see Fig. 2). Shifting cells from glucose to galactose media also induced mitochondrial localization of NIK and DRP1 in COS-7 cells (Supplementary Fig. 2c–g), demonstrating that these results are not limited to GBM cells. These data show that glucose starvation and forced reliance on oxidative metabolism induce NIK- and DRP1-dependent mitochondrial fission, as well as NIK-dependent mitochondrial localization of DRP1.

### NIK promotes cell survival by increasing mitochondrial SRC in response to metabolic shift to OXPHOS

**Fig. 4:**
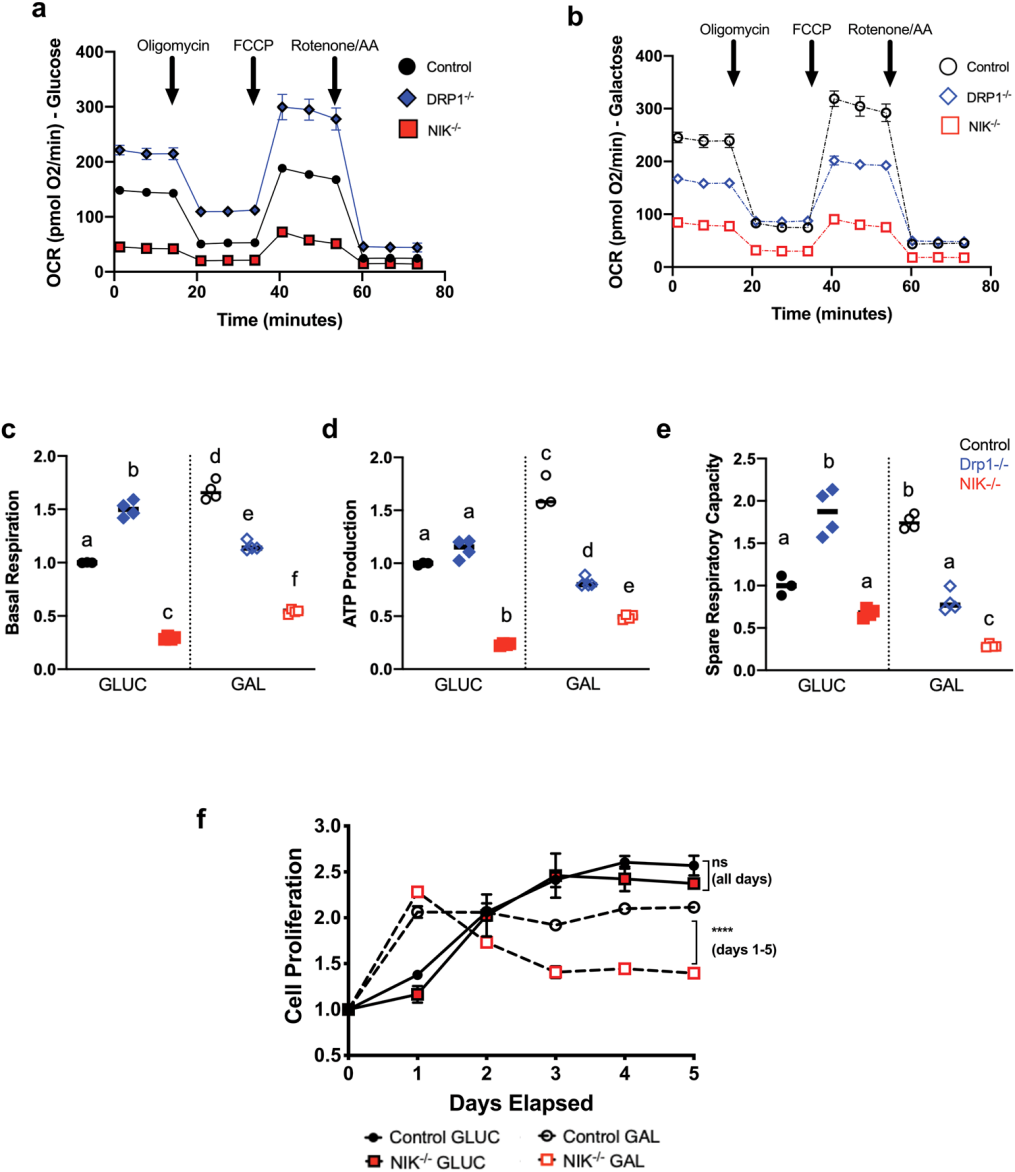
NIK promotes cell survival by increasing mitochondrial spare respiratory capacity in response to forced reliance on OXPHOS. **a**, **b** Oxygen consumption rate (OCR) was measured using the Seahorse Mito Stress test with control cells (see Fig. 2b), DRP1^−/−^ and NIK^−/−^ cells that were cultured in **a** 18 mM glucose (GLUC), or **b** 18 mM galactose (GAL) media. Data shown are the mean ± SD of ≥3 replicates for each cell type in each condition and are representative of at least three independent experiments. OCR values were normalized to cellular DNA content (DRAQ5 relative fluorescence units (RFUs)). **c–e** Individual mitochondrial function parameters were calculated from the data shown in **a** and **b** and shown as fold change compared to control cells in GLUC. Data for control cells was taken from Fig. 2b. **c** Basal respiration. Different letters indicate statistical significance using one-way ANOVA with Tukey post-hoc test. All comparisons have *p* < 0.0001 except “a vs e” (*p* < 0.05) and “b vs d” (*p* < 0.01). **d** ATP production. All statistical comparisons have *p* < 0.0001 except “a vs d” (*p* < 0.05), “c vs f” (*p* < 0.001). **e** Spare respiratory capacity. All statistical comparisons have *p* < 0.0001 except “a vs c” (*p* < 0.05). **f** Representative MTS Proliferation assay. Control and NIK^−/−^ cells were cultured in GLUC or GAL for the indicated times. Proliferation is represented as fold change compared to day 0. Data are representative of three different experiments and are shown as mean ± SD (*n* = 3) with two-way ANOVA followed by Tukey post-hoc test (****p < 0.0001).

Thus far, our data suggest that NIK is important for the adaptive response of GBM cells to bioenergetic stress whereby cells sense and respond to a forced metabolic switch to OXPHOS by increasing mitochondrial NIK localization and mitochondrial fission. To compare the effects of altering cellular bioenergetic demand on GBM cell metabolism, we compared metabolic changes under glucose and galactose growth conditions. Under high glucose (18 mM) conditions, OCR is increased in DRP1^−/−^ cells compared to control cells (Fig. 4a), consistent with previous studies describing a role for mitochondrial fusion in promoting OXPHOS^7,26^. In contrast, NIK^−/−^ GBM cells exhibit significantly lower OCR compared to control cells (Fig. 4a and Supplementary Fig. 1e). When cells were cultured in galactose, control cells increased OCR and basal respiration to compensate for reduced glycolysis, whereas DRP1^−/−^ cells exhibited decreased OCR and basal respiration (Fig. 4b, c). NIK^−/−^ cells slightly increased OCR and basal respiration in galactose vs. glucose, but to a much lower extent compared with control and DRP1^−/−^ cells (Fig. 4b, c and Supplementary Fig. 6a). Consistent with increased OCR and basal respiration, control cells also exhibited increased mitochondrial ATP production and SRC in response to the increased energy demand caused by growth in galactose (Fig. 4d, e and Supplementary Fig. 6b, c). However, mitochondrial ATP production and SRC were both significantly reduced in galactose-cultured DRP1^−/−^ and NIK^−/−^ cells compared to galactose-cultured control cells (Fig. 4d, e). Interestingly, NIK^−/−^ cells exhibited significantly decreased SRC in galactose compared with glucose, indicating that they lack the metabolic reserves required for adaptation to bioenergetic stress (Fig. 4e and Supplementary Fig. 6c). Indeed, when cells were shifted to galactose to force OXPHOS-dependent growth, NIK^−/−^ cells exhibited significantly reduced proliferation compared with control cells (Fig. 4f). These results demonstrate that NIK is required in GBM cells to adapt metabolically to changes in bioenergetic demands, thereby promoting cell growth.

### NIK regulation of mitochondrial oxidative metabolism and cell survival is independent of IKK and NF-κB

**Fig. 5:**
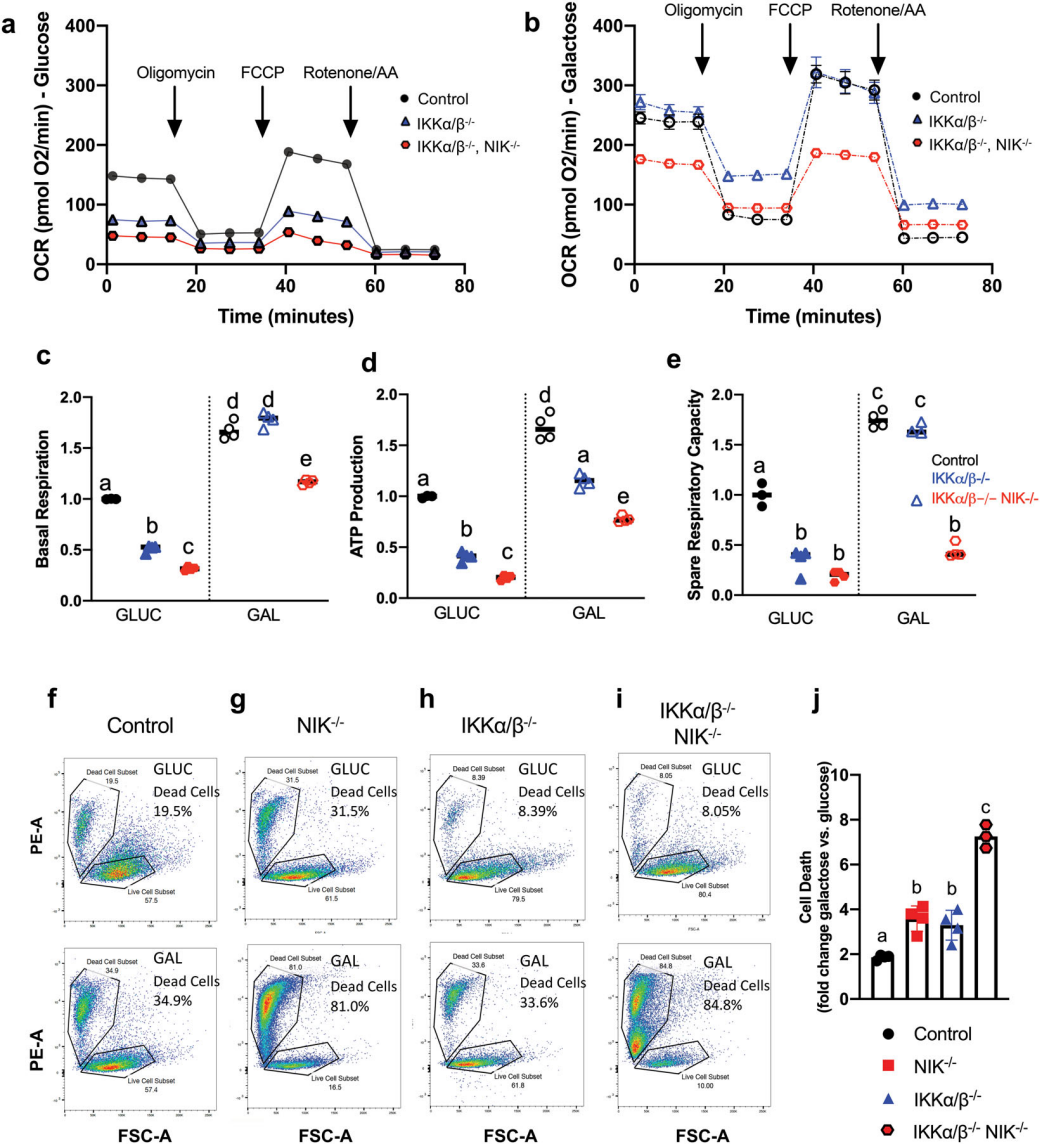
NIK regulation of mitochondrial metabolism and cell survival is independent of IKK. **a**, **b** Oxygen consumption rate (OCR) was measured using the Seahorse Mito Stress test with control cells (see Figs. 2b and 4a, b), IKKα/β^−/−^ cells and IKKα/β^−/−^, NIK^−/−^ lines that were cultured in 18 mM glucose (GLUC) (**a**) or 18 mM galactose (GAL) media (**b**). Data shown are the mean ± SD of ≥3 replicates for each cell type in each condition and are representative of at least three independent experiments. OCR values were normalized to cellular DNA content (DRAQ5 relative fluorescence units (RFUs)). **c–e** Individual mitochondrial function parameters were calculated from the data shown in **a** and **b** and shown as fold change compared to control cells in GLUC. Data for control cells was taken from Fig. 2b. **c** Basal respiration. Different letters indicate statistical significance using one-way ANOVA with Tukey post-hoc test. All comparisons have *p* < 0.0001 except “a vs e” (*p* < 0.01), “b vs c” (*p* < 0.001). **d** ATP production. All statistical comparisons have *p* < 0.0001 except “a vs e” (*p* < 0.01), and “b vs c” (*p* < 0.01). **e** Spare respiratory capacity. All statistical comparisons have *p* < 0.0001 except “a vs b” (*p* < 0.01), “a vs c” (*p* < 0.05). **f**–**i** Representative Flow cytometry analysis of cell death using propidium iodide staining. Indicated BT25 GBM cells were grown for 48 h in either 18 mM glucose (upper panels) or galactose media (lower panels). **j** Fold change in cell death induced by shifting from GLUC to galactose media for 48 h (% dead cells in galactose / % dead cells glucose). Different letters indicate statistical significance using one-way ANOVA with Tukey post-hoc test. For “a vs b” *p* < 0.05, “b vs c” *p* < 0.0001; *n* = 3–4 independent experiments, *n* ≥ 10,000 cells per condition. Error bars indicate mean ± SD.

Although NIK is well established as a positive regulator of the inhibitor of kB kinase (IKK) complex, which is required for activation of NF-κB signaling, IKK-independent functions for NIK have also been described^20,27^. Thus, we sought to determine whether NIK-dependent regulation of mitochondrial metabolism is mediated by IKK and downstream NF-κB signaling. Using CRISPR-Cas9 genome editing, we generated GBM cells lacking both IKKα and IKKβ (IKKαβ^−/−^) and verified that they were indeed deficient in downstream NF-κB signaling (Supplementary Fig. 4a, b). Under high-glucose growth conditions, IKKα/β^−/−^ cells exhibited significantly impaired OCR (Fig. 5a), consistent with previously described roles for NF-κB in regulating mitochondrial metabolism^28,29^. This OCR defect was even more pronounced in triple knockout cells lacking IKKα, IKKβ, and NIK (IKKα/β^−/−^ NIK^−/−^) (Fig. 5a). Interestingly, despite defective OCR in high glucose, IKKα/β^−/−^ cells behaved similarly to control cells when shifted to galactose as they were able to significantly increase OCR, basal respiration, mitochondrial ATP production, and SRC (Fig. 5b–e and Supplementary Fig. 6d–f). In contrast, while IKKα/β^−/−^ NIK^−/−^ triple knockout cells increased OCR, basal respiration, and ATP production when shifted from glucose to galactose (Supplementary Fig. 6d, e), these metabolic parameters were not increased to the same overall extent as galactose-cultured control or IKKα/β^−/−^ cells (Fig. 5b–d). Moreover, SRC was most significantly impaired in IKKα/β^−/−^ NIK^−/−^ triple knockout cells (Fig. 5e and Supplementary Fig. 6f). We next examined whether NIK regulated cell survival in response to metabolic shift to OXPHOS. Control cells exhibited a small increase in cell death when shifted from glucose to galactose (Fig. 5f and Supplementary Fig. 5c), which was increased in NIK^−/−^ and IKKα/β^−/−^ cells (Fig. 5g, h). However, the highest level of galactose-induced cell death occurred in IKKα/β^−/−^ NIK^−/−^ cells (Fig. 5i), and the effect was additive compared to NIK^−/−^ or IKKα/β^−/−^ cells alone (Fig. 5j), suggesting independent contributions. Taken together, these results demonstrate that NIK controls metabolic adaptation to bioenergetic stress and promotes cell survival independently of IKK/NF-κB.

### NIK phosphorylates DRP1 at serine 616

**Fig. 6:**
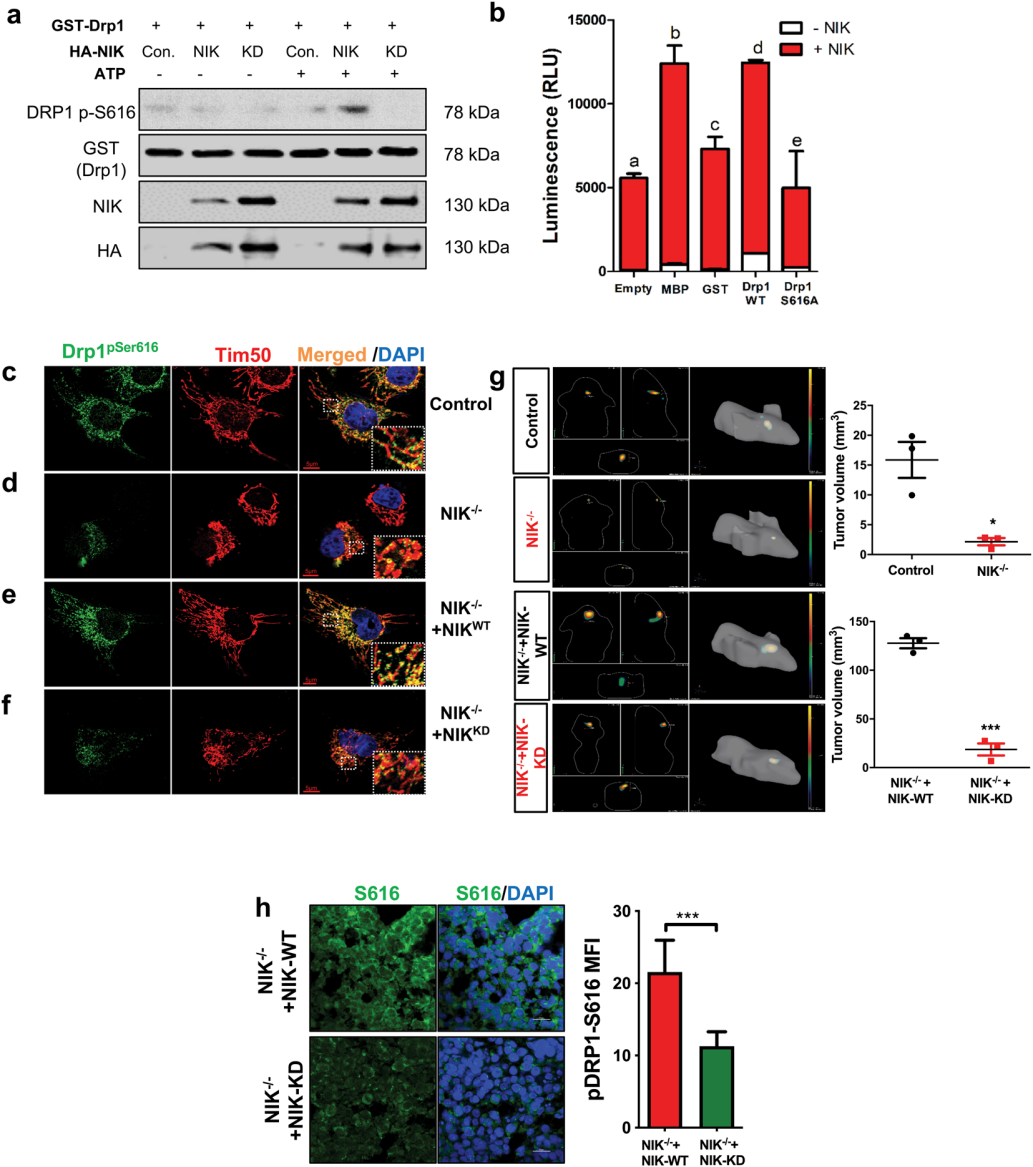
NIK phosphorylates DRP1 at serine 616. **a** Kinase assays were performed using 293T cells transfected with HA-NIK wild-type (NIK) or kinase-dead mutant K429A/K430A (KD) expression vectors. NIK constructs were immunoprecipitated with anti-HA beads, and the immunocomplexes were incubated with purified GST-DRP1^518–736^ in the absence or presence of 20 μM ATP for 15 min at 37 °C. Kinase reactions were subjected to SDS-PAGE and probed with the indicated antibodies. **b** ADP-Glo kinase assay (Promega) was performed using 25 ng of purified recombinant human NIK protein (325aa-end) incubated with either GST alone, GST-DRP1^518–736^ (DRP1 WT) or GST-DRP1^518–736,S616A^ (DRP1-S616A), or myelin binding protein (MBP, positive control). White overlay indicates level of NIK autophosphorylation. Different letters represent statistically significant differences (for “a vs b” and “a vs d”, *p* < 0.001; for “b vs c”, *p* < 0.01, for “c vs d”, *p* < 0.05; one-way ANOVA, *n* = 4). **c**–**f** BT25 control, BT25-NIK^−/−^, BT25-NIK^−/−^+NIK^WT^ rescue, and BT25-NIK^−/−^+NIK^KD^ rescue cells were immunostained using a DRP1-P616-specific antibody (Green), Tim50 (red), and DAPI (blue). Scale bar, 5 μm. Areas within dotted white squares are enlarged and shown on the right. **g** 3D rendering by In Vivo Imaging System (IVIS) of DiD labeled BT25 control, NIK^−/−^, NIK^−/−^+NIK-WT, and NIK^−/−^+NIK-KD cells at day 30. Tumor size (right graphs) was calculated at 30 days post injection of each cell type (*n* = 3 per cell type). Tumor volume was calculated by the formula: tumor volume [mm3] = (length [mm]) × (width [mm]) × (height [mm]) × (*π*/6) at 30 days post injection (*n* = 3, respectively). Data represent average tumor volume ± S.E.M.; ***p < 0.001, unpaired Student’s *t*-test. Statistical significance was determined by Student’s *t* test, * indicates *p* < 0.05, *** indicates *p* < 0.001. **h** (Left) Immunofluorescence staining of NIK^−/−^+NIK-WT and NIK^−/−^+NIK-KD tumors with phospho-DRP1 S616 antibody, (right) fluorescence intensity of phospho-DRP1 staining using *n* = 10–12 fields of view per cell type. Significance was determined by Student’s *t* test; *** indicates *p* < 0.001.

It is well established that phosphorylation of DRP1-serine 616 is critical for mitochondrial fission^10,30,31^, and we previously demonstrated GBM cells lacking NIK exhibited reduced DRP1 phosphorylation at S616 (pDRP1-S616)^20^. Given the findings that NIK and DRP1 colocalize dynamically at the mitochondrial constriction sites during fission, and that NIK regulates mitochondrial metabolism and OXPHOS-dependent cell survival independently of IKK, we sought to determine whether NIK directly phosphorylated DRP1. Using in vitro kinase assays, we observe that DRP1-S616 was phosphorylated by NIK^WT^, but not a kinase-dead, S429A/S430A NIK^KD^ mutant construct that lacks kinase activity (Fig. 6a and Supplementary Fig. 3). Purified, recombinant NIK protein (amino acids 325–947) was capable of phosphorylating GST-DRP1 WT, but not a GST-DRP1-S616A mutant (Fig. 6b). Immunofluorescence experiments revealed abundant mitochondrial pDRP1-S616 signal that was significantly decreased in NIK^−/−^ cells (Fig. 6c, d), and ectopic expression of wild-type NIK, but not NIK^KD^ mutant, in NIK^−/−^ cells restored pDRP1-S616 signal and mitochondrial localization (Fig. 6e, f). Additionally, NIK^−/−^ cells expressing a NIK kinase dead mutant formed smaller orthotopic intracranial tumors with lower pDrp1-S616 immunostaining compared with NIK^−/−^ cells rescued with wild-type murine NIK (mNIK) (Fig. 6g, h). These results demonstrate that NIK regulates DRP1 phosphorylation, in vitro and in vivo.

### Constitutively active DRP1 rescues oxidative metabolism and tumorigenic potential in NIK^−/−^ GBM cells

**Fig. 7:**
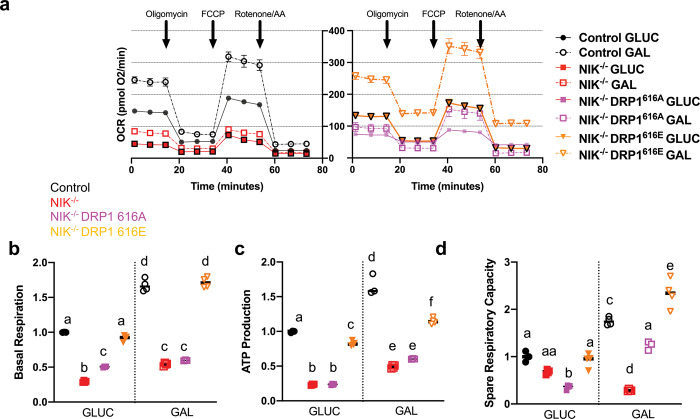
Constitutively active DRP1-S616E restores oxidative metabolism of NIK-deficient GBM cells. **a** Oxygen consumption rate was determined using the Seahorse Mito Stress test with control and NIK^−/−^ cells (see Figs. 2b, 4a, b), as well as NIK^−/−^ cells ectopically expressing DRP1-S616E that were cultured in 18 mM glucose (GLUC) or 18 mM galactose (GAL) media. Data depict the mean of ≥3 replicates per each cell type. **b**–**d** Individual mitochondrial function parameters were calculated from the data shown in **a** and shown as fold change compared to control cells in GLUC. **b** Basal respiration. Different letters indicate statistical significance using one-way ANOVA with Tukey post-hoc test. All comparisons have *p* < 0.0001. **c** ATP production. All statistical comparisons have *p* < 0.0001 except “a vs f” (*p* < 0.001). **d** Spare respiratory capacity. All statistical comparisons have *p* < 0.0001 except “a vs aa” (ns), “aa vs b” (*p* < 0.001), “aa vs d” (*p* < 0.05) “a vs c” (*p* < 0.001), and “c vs e” (*p* < 0.001).

To examine whether DRP1 could rescue the metabolic and tumorigenic potential in the absence of NIK, we performed gain-of-function studies using a DRP1 mutant with a serine 616 glutamic acid (S616E) substitution, to mimic phosphorylation and induce mitochondrial fission, as well as the corresponding DRP1-S616A substitution, which cannot be phosphorylated, and inhibits fission^32,33^ (Supplementary Fig. 5a). Seahorse Analyzer Flux data revealed that expression of the DRP1-S616E phosphomimetic in NIK^−/−^ GBM cells restored OCR and basal respiration (Fig. 7a, b), ATP production (Fig. 7c), and SRC (Fig. 7d) cultured in galactose, comparable to control cells (see also Supplementary Fig. 6). Importantly, DRP1-S616E did not activate IKK to induce p100 processing to p52 (Supplementary Fig. 5a).

**Fig. 8:**
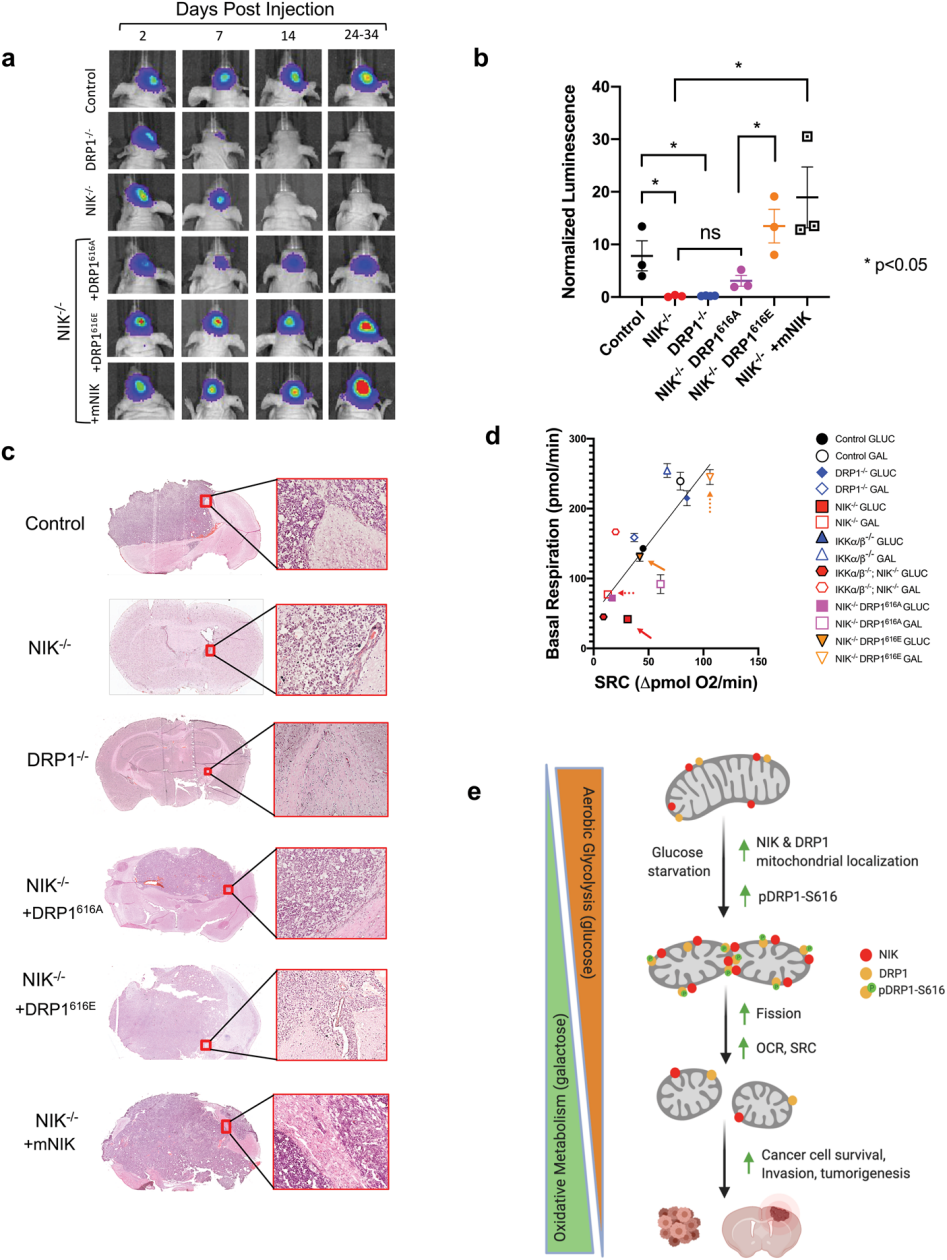
Constitutively active DRP1 rescues oxidative metabolism and tumorigenic potential in NIK^−/−^ GBM cells. **a** Representative cropped images from IVIS imaging showing luminescence of individual mice pictured here over the course of 35 days, *n* ≥ 3 mice per cell type. Mice were imaged at regular intervals with the exception of the final endpoint since mice with large tumor growth required euthanasia at earlier timepoints. Mice harboring DRP1^−/−^, NIK^−/−^, and NIK^−/−^+DRP1^616A^ tumors were last imaged at day 35 and mice harboring NIK^−/−^+DRP1^616E^ and NIK^−/−^+DRP1^616E^+mNIK tumors were last imaged at days 28 and 24, respectively. **b** Graph depicting fold change luminescence on day 28 from fold change on day 7, with the exception of NIK^−/−^+mNIK which is fold change from day 21. **c** Representative images of tumors and histology (pictured as enlargements of red box inset) in control, NIK^−/−^, DRP1^−/−^, NIK^−/−^ DRP1^616A^, NIK^−/−^ DRP1^616E^, and NIK^−/−^+mNIK. **d** Composite graph plotting basal respiration vs mean spare respiratory capacity (SRC) (*n* ≥ 3 replicates per each cell type). Solid arrows indicate glucose condition and dotted arrows indicate galactose condition for NIK^−/−^ and NIK^−/−^ DRP1^616E^ cell lines (red and orange, respectively). **e** Model: NIK and DRP1 mitochondrial localization increase upon glucose starvation and switch to galactose media resulting in transition from aerobic glycolysis to reliance on oxidative metabolism. NIK regulates DRP1 phosphorylation (pDRP1-S616), increases mitochondrial fission, OXPHOS, and spare respiratory capacity (SRC), leading to increased cancer cell survival, invasion, and tumorigenic potential.

Next, we tested whether constitutively active DRP1-S616E was able to rescue NIK^−/−^ cell phenotypes. We observed that DRP1-S616E rescued the cell death observed when NIK^−/−^ cells were shifted from glucose to galactose (Supplementary 5b). Three-dimensional (3D) collagen invasion assays demonstrated that NIK was required for GBM invasion (Supplementary Fig. 7a), as we have previously described^20,34^. Expression of the DRP1-S616E phosphomimetic mutant increased the invasion potential of NIK^−/−^ cells to a much greater extent than DRP1-S616A (Supplementary Fig. 7a). Orthotopic xenografts of BT25 cells demonstrated that loss of DRP1 or NIK significantly attenuated intracranial GBM tumor growth in vivo (Fig. 8a, b). Similar to the 3D invasion assays, overexpression of DRP1-S616E restored the growth of NIK^−/−^ cells to levels comparable to NIK^−/−^ cells rescued with wild-type murine NIK (NIK^−/−^ +mNIK) (Fig. 8a–c). Finally, to gain an overall assessment of the oxidative metabolism and metabolic fitness across the NIK^−/−^, IKK^−/−^, and DRP1^−/−^ mutants cell lines, we plotted the relationship between SRC and OCR. This analysis demonstrated that NIK^−/−^, IKKα/β^−/−^, and IKKα/β^−/−^ NIK^−/−^ triple knockout cells cultured in glucose and NIK^−/−^ in galactose are least oxidative, whereas DRP1^−/−^ cells in glucose as well as control, IKKαβ^−/−^, and NIK^−/−^ +DRP1-S616E cells cultured in galactose are the most oxidative (Fig. 8d). Taken together, these results are consistent with a model in which NIK controls mitochondrial metabolism to promote GBM cell invasion and survival through DRP1-mediated regulation of mitochondrial dynamics, independently of IKK (Fig. 8e).

## Discussion

We previously reported that a pool of NIK protein localizes to mitochondria in cancer cells under basal, steady-state conditions, where it promotes mitochondrial fission and cell migration^20^. In this study, we have demonstrated a role for NIK in regulating adaptation to growth conditions shifting GBM cells from glycolytic metabolism in high glucose to forced reliance on mitochondrial metabolism in galactose. We found that NIK association with mitochondria increases under glucose deprivation to promote DRP1 mitochondrial association, fission, and oxidative metabolism, supporting cell survival and tumorigenesis. Specifically, we observed that NIK is required for GBM cells to enhance mitochondrial SRC, which is used to generate extra ATP via OXPHOS in response to sudden increases in energy demands and is, therefore, a strong indicator of metabolic fitness.

Although several components of the NF-κB/IKK signaling pathway, including IKKα, IKKβ, have been putatively identified in mitochondria^35–38^, there are limited data on the function of these proteins in this organelle. NF-κB and its upstream regulatory proteins, including NIK, were previously shown to control energy homeostasis and metabolic adaptation to glucose starvation by transcriptional upregulation of genes controlling mitochondrial respiration^28^. Here, we provide evidence that NIK can regulate mitochondrial metabolism and adaptation to bioenergetic stress independently of NF-κB/IKK. First, GBM cells lacking IKKα/β and NIK had an additive impact on cell death compared with cells lacking IKK or NIK alone (Fig. 5), demonstrating that they are not redundant. Second, while cells lacking either IKKα/β or NIK both exhibited impaired oxidative metabolism in high glucose, only NIK-deficient cells were unable to increase mitochondrial OCR and SRC under glucose deprivation. Third, NIK, but not IKKα/β, directly phosphorylates DRP1 at S616, a site well-known to be required for its fission-promoting activity (Fig. 6 and Supplementary Fig. 5). Notably, a fission-promoting, phosphomimetic DRP1-S616E, but not a DRP1-S616A mutant, rescued oxidative metabolism and adaptation to galactose in NIK^−/−^ cells without activating downstream IKK/NF-κB signaling (Figs. 7, 8 and Supplementary Fig. 5). The results support our previous observation that NIK regulation of mitochondrial dynamics does not require IKKα/β or NF-κB^20^. Furthermore, these findings are consistent a recent observation by Nagdas et al. that DRP1^−/−^ cells are more sensitive to galactose growth conditions that force reliance on OXPHOS^39^. Taken together, these results suggest that DRP1-dependent mitochondrial fission is critical for increasing oxidative metabolism to promote tumor growth.

We observed that NIK^−/−^ and DRP1^−/−^ cells exhibited some distinct metabolic responses. For example, in high glucose, NIK^−/−^ GBM cells had impaired oxidative metabolism, but also exhibited increased glycolysis (Supplementary Fig. 7), and this compensatory effect likely explains why NIK^−/−^ cell proliferation is not significantly impaired under high-glucose conditions (Fig. 4f). On the other hand, in high glucose, DRP1^−/−^ cells with hyperfused mitochondria displayed increased OCR (Fig. 4a). This result is consistent with many studies in yeast and mammalian cells showing that mitochondrial fusion is important for regulation of OXPHOS^40–42^. Under galactose-induced metabolic stress, DRP1^−/−^ and NIK^−/−^ cells were both impaired in their ability to increase OCR to the extent of control cells. However, unlike NIK^−/−^ cells, DRP1^−/−^ cells did not exhibit increased glycolysis (Supplementary Fig. 8). Indeed, while the DRP1-S616E mutant fully rescued NIK^−/−^ OXPHOS defects, it had an inhibitory effect on glycolysis (Supplementary Fig. 8). In contrast, DRP1 was shown to promote glycolysis in KRas-driven pancreatic cancer^39^, suggesting that DRP1 regulation of glycolysis may be cell-context dependent and controlled through distinct posttranslational DRP1 modifications. Additionally, although we clearly show that NIK can regulate mitochondrial functions in the absence of IKK, given the impairment of OCR in IKKα/β^−/−^ cells under high glucose, it is likely that NIK can also impact metabolism through IKK/NF-kB signaling under specific growth conditions.

Mitochondria undergo dynamic morphological changes in response to environmental stress and nutrient availability has been shown to dictate mitochondrial morphology^7,21,43^. Our findings are consistent with previous studies demonstrating that nutrient starvation induces mitochondrial fission^44–46^ and suggest that metabolic regulation of mitochondrial dynamics may be determined by NIK activity to promote metabolic reprogramming to OXPHOS and drive invasive growth. Indeed, analysis of RNA-seq data from the IVY Glioblastoma Atlas^47^ revealed that genes regulating mitochondrial dynamics, including DRP1, as well as mitochondrial respiratory complexes are more highly expressed at the tumor leading edge compared to the tumor core (Supplementary Fig. 7). In contrast, genes regulating hypoxia are enriched in the tumor core. Future experiments are needed to address whether NIK is involved in mediating adaptive responses to other stresses that are characteristically observed in GBM, such as hypoxia and nutrient deprivation.

In summary, our studies identify NIK as a critical regulator of mitochondrial fission and metabolic adaptation of cancer cells, allowing them to meet continuously changing bioenergetic demands and survive harsh tumor microenvironments encountered during tumor growth and metastasis. Our findings strongly indicate that NIK inhibition may be a critical component of therapeutic modalities that target vulnerabilities in GBM metabolism to improve patient survival.

## Materials and methods

### Cell culture

BT25 and BT114 patient-derived cells were obtained as previously described^48^. Cells were maintained as spheroids in Neural Stem Cell (NSC) medium containing advanced DMEM/F-12, 1× B-27 supplement minus vitamin A, 1× Glutamax, 25 ng/ml EGF, 25 ng/ml bFGF, and 1× Pen/Strep (Life Technologies). Tumorspheres were not cultured beyond ~100 μm. 293T and COS-7 cells were obtained from ATCC and cultured in DMEM medium with 10% FBS and 1× Pen/Strep. All cells were cultured at 37 °C with 95% humidity and 5% CO_2_. All cell lines are routinely tested for mycoplasma and authenticated by STR fingerprinting.

### Immunofluorescence staining

A total of 2 × 10^4^ cells were seeded in collagen-coated (40–50 μg/ml) 8-well chamber slides (Ibidi, #80827, Munich, Germany) or on coverslips in the presence of 1% FBS and allowed to adhere overnight. Cells were fixed with 4% paraformaldehyde, permeabilized for 20 min with 0.3% Triton X-100 in PBS, and blocked for 30 min at room temperature with 5% serum corresponding to secondary antibody host. Cells were incubated overnight in 0.1% Triton X-100, 1% BSA in PBS at 4 °C with either the primary antibody phospho-DRP1 S616 (Cell Signaling Technology, 4494), Tim50 (Santa Cruz, sc-393678), or Tom20-AF488 (Abcam, ab205486). Cells were then incubated in 1% BSA for 1 h at room temperature with secondary antibodies Alexa Fluor 488 or Alexa Fluor 594 from Life Technologies.

### Live cell imaging

BT25 cells and COS-7 cells were plated on 8-well chamber slides with a glass bottom (Ibidi) and then were transfected with Lipofectamine 3000 (Invitrogen) with constructs expressing GFP-NIK, mCherry-Drp1 (mch-Drp1), and mito-BFP. After 18 h, time-lapse images were acquired every 5 s for 3 min on a Nikon TI A1R inverted confocal microscope with 60× Plan-Apochromat lenses and Stage-top incubator system. Random fields of mitochondria were imaged (*n* > 19 cells each) and analyzed using NIS-Elements imaging software (Nikon, Japan). The diffuse cytosolic images were threshold-adjusted by subtracting background signal in the cytosol. For analysis of COS-7 cells under metabolic switching conditions, cells were seeded at a density of 50,000 cells into a collagen-coated (50 μg/ml), 8-chamber slide (Ibidi) with 1% FBS. Twenty-four hours later, cells were transfected with GFP-NIK, mCherry-DRP1, Mito-BFP (40 ng each) using lipofectamine (Lipofectamine 3000, Invitrogen); 24–48 h after transfection, cell media was either switched to media containing 25 mM galactose (without glucose) or media containing 25 mM glucose. Cells were live imaged as described above. Correlation analysis was done on RBG images using NIS Elements software. Mander’s correlation coefficient of the pixel overlap was measured in individual frames from enhanced video.

### Subcellular fractionation

Subcellular fractionation was performed as previously described^49^. In brief, cultured cells were washed twice with ice-cold PBS and scraped in 2 ml of ice-cold PBS. After removing supernatants following centrifugation at 800 × *g* for 5 min, pellets were resuspended and homogenized in homogenization buffer (10 mM HEPES-KOH, pH 7.5, 0.25 M sucrose) by passing a 27-gauge needle ten times. Post-nuclear supernatants were collected by centrifugation at 1000 × *g* for 10 min and then, heavy membrane fraction was separated from the resultant supernatant by centrifugation at 8000 × *g* for 10 min at 4 °C. The pellets of heavy membrane fraction, mitochondria-enriched fraction were washed with buffer and solubilized in 100 μl of solubilization buffer (50 mM Tris-HCI [pH 8.8], 5 mM EDTA, 1% SDS). The remaining supernatants as cytosol fraction were centrifuged again at 16,000 × *g* for 10 min at 4 °C to remove any remaining insoluble membranes.

### Immunoblot assays

Cells were lysed in RIPA lysis buffer (Pierce, #89900, Rockford, lL) with protease/phosphatase inhibitor cocktail (Thermo Scientific). Equal amounts of protein were mixed with NuPage 4X LDS sample buffer (Invitrogen, NP0008) containing reducing agent and denatured at 100 °C for 7 min. Proteins were separated on 8–12% SDS-PAGE and transferred to nitrocellulose membranes (Bio-Rad, #162-0115). The membranes were blocked for 1 h with 5% non-fat dry milk in 0.1% Tween-20/TBS (TBST) or Odyssey blocking buffer (LI-COR Biosciences, 927-40000) and incubated with primary antibodies for either IKKα (EMD Millipore, OP133), phospho IKKα/β (Ser176/180) (Cell Signaling Technology, 2697S), NIK (Cell Signaling Technology, 4994S), HA-tag (Cell Signaling Technology, 3724S), V5-tag (Cell Signaling Technology, 13202S), GST (Santa Cruz Biotechnology, sc-138), NFKB2 (Cell Signaling Technology, 4882S), GAPDH (Santa Cruz Biotechnology, sc-365062), β-actin (Santa Cruz Biotechnology, sc-69870), Tom20 (Cell Signaling Technology, 52406S), TOM70 (Santa Cruz Biotechnology, sc-390545), HSP60 (Cell Signaling Technology, 12165S), OPA1 (Cell Signaling Technology, 80471S), DRP1 (Cell Signaling Technology, 8570S), phospho-DRP1 S616 (Cell Signaling Technology, 4494S) diluted in blocking buffer at 4 °C overnight. After washing in TBST, membranes were incubated with goat anti-rabbit IRDye800CW (LI-COR Biosciences), goat anti-mouse IRDye680 (LI-COR Biosciences), or goat anti-rabbit HRP conjugate (Thermo Scientific) diluted in blocking buffer and incubated for 1 h at room temperature. The blots were washed with TBST and developed using Chemiluminescent HRP Substrate (EMD Millipore, WBKLS0100) for detection of HRP or an Odyssey Infrared Imaging system (LI-COR Biosciences) for detection of IR fluorescent dyes.

### Proliferation and cell survival assay

BT25 cells were transitioned to low glucose media + galactose as follows: After initial culturing for 5–7 days in NSC media, cells were dissociated and seeded at a density of 5 × 10^5^ cells and cultured for 5–7 days sequentially in DMEM/F12 media (US Biological Life Sciences, D9807-02, Salem, MA) containing 18 mM d-Glucose, then DMEM/F12 media containing 5 mM d-Glucose, and finally 18 mM d-Galactose, 2 mM d-Glucose. All media contained 1 mg/l insulin (Gibco, 12585014). For MTS (Cell Titer AQ_ueous_ One Solution Cell Proliferation Assay, G3581, Promega), cells transitioned to low glucose/galactose were seeded at a density of 1 × 10^4^ cells on collagen-coated 96-well plates. Absorbance readings (490 nm) were then taken at indicated times using the Victor X3 microplate reader and normalized to day 0. For propidium iodide (PI) cell death flow cytometry analysis, cells were transitioned to galactose as described above. After culturing for 48 h in either DMEM/F12 NSC media containing 18 mM glucose or 18 mM galactose, cells were dissociated with Accutase, stained with PI according to the manufacturer’s instructions (ThermoFisher Scientific, Alexa Fluor 488 Annexin V Dead Cell Apoptosis kit, V13241), and analyzed using a Fortessa X-20 flow cytometer. Cell death was measured as fold change % dead cells in galactose divided by % dead cells in glucose, with *n* ≥ 3 replicates per condition.

### Plasmids

For NIK overexpression, mouse (mNIK) cDNA was cloned into pLenti6-V5-DEST (Addgene, Cambridge, MA) using the GATE-WAY^TM^ Cloning System (Invitrogen). Luciferase (Promega) coding sequences were subcloned into pLenti6-v5-DEST (Invitrogen) and served as a control vector for NIK overexpression. For GFP-NIK construct, GFP was fused to the N-terminal of full-length human NIK (modified from Addgene, Cambridge, MA plasmid #27554). For DRP1-S616E overexpression, DRP1 616E was created by point mutagenesis using Q5™ Site Directed Mutagenesis Kit (NEB) and the following oligos: DRP1 616E up: TATGCCAGCCGCTCCACAAAAAG. DRP1 616E dn: ATTGGAATGGGTTTTGATTTTTC. The mutated construct was then cloned into pLenti6-V5-DEST (Addgene) using the GATE-WAY™ Cloning System (Invitrogen). For DRP1 S616A construct, which additionally harbors a S637E mutation, we utilized the same strategy as with 616E using following oligos: DRP1 616SA up: TATGCCAGCCGAGCCACAAAAAG, DRP1-S616SA dn: ATTGGAATGGGTTTTGATTTTTC. DRP1 S637SE up: ACGAAAACTAgctGCTCGGGAAC, DRP1-S637SE dn: GCAACAGGAACTGGCACATC.

### CRISPR-Cas 9 gene knockout

BT25 cells were transduced with a mixture of Lenti-CrispR-v2 carrying three gRNAs for each target. The gRNA sequences for human NIK and DRP1 are previously described^20,50^. The sequences for IKKα are: gIKKa1 ACAGACGUUCCCGAAGCCGC, gIKKa2 ACGUCUGUCUGUACCAGCAU, gIKKa3 UUCUGGAGGAGAUCUCCGAA. The sequences for IKKβ are: gIKKb1 GAUUUGGAAAUGUCAUCCGA, gIKKb2 UCAGCCCCCGGAACCGAGAG, gIKKb3 CCUUCCGGAGAUCUCCUCCU. Loss of NIK, Drp1, IKKα, and IKKβ expression was confirmed by immunoblot, qPCR, and/or immunofluorescence microscopy analysis of puromycin-resistant cells. For BT25-NIK^−/−^, DRP1^−/−^, IKKα/β^−/−^ cells, single colony cells were isolated by serial dilution. All experiments were repeated with at least two knockdown clones.

### Lentivirus production

Twenty-four micrograms of lentiviral plasmids and 72 μg of polyethyleneimine were used to transfect 293T cells. After 3 days of transfection, viral supernatant was harvested and filtered through a 0.45-µM syringe filter. After filtration, viral particles were concentrated 20-fold to 500 µl using Lenti-X Concentrator (Clontech, #631231, Mountain View, CA), and 100 µl of concentrated virus was used to infect cells. Stably transduced cells were selected for 72 h in medium containing 0.6 μg/ml puromycin or 3 μg/ml blasticidin (Invitrogen).

### Metabolic assays

Metabolic activity was analyzed using a Seahorse XFe96 Analyzer (Agilent, Santa Clara, CA). Metabolic shifting was performed as described above for proliferation assays. Thirty-thousand cells per well were plated in the collagen-coated Aligent Seahorse XFe96 microplates and left to settle overnight before analysis with the FluxPak kits according to company standards. Mitochondrial Stress Tests were conducted according to the manufacturer’s guidelines. For the assay base, media was supplemented with either 18 mM glucose (Sigma, G7021, St. Louis, MO) or 18 mM galactose (Sigma, G0750, St. Louis, MO), 2 mM glutamine (Sigma, G85420, St. Louis, MO), and 1 mM pyruvate (Gibco). Inhibitors were used at the following concentrations: 1 μM oligomycin A (Sigma, 75351, St. Louis, MO), 1 μM FCCP (Sigma, C2920, St. Louis, MO), and mixture of 0.5 μM rotenone (Enzo Life Sciences, Farmingdale, NY, ALX-350-360) and 0.5 μM antimycin A (Sigma, A8674, St. Louis, MO). After Seahorse analysis, DNA content was measured using DRAQ5 staining (Thermo Fisher Scientific, 50-712-282) for normalization. Analyses were conducted using Seahorse Wave Controller Software v2.6 and XF Report Generators (Agilent Technologies). For glycolysis stress tests, cells were plated on collagen-coated Seahorse XFe96 plates overnight in 18 mM galactose + 2 mM glucose and then glucose-starved for 1 h in Seahorse DMEM base media before analysis. The BT25 control 18 mM glucose and 18 mM galactose data points were used as references for Figs. 2, 4, 5, and 7. Each experiment contained 3-4 replicates per condition and was repeated ≥3 independent times.

### Single organelle analysis of mitochondria by flow cytometry

For single organelle flow cytometry analysis^51^, BT25 NIK^−/−^ +mNIK-v5 cells were plated in 6 cm dishes in Advanced DMEM/F12 complete NSC media with 1% FBS and grown to roughly 85–90% confluence before being switched to 18 mM glucose DMEM/F12 with B27 supplement, EGF, bFGF, 10 mg/l insulin, and 1% FBS. For galactose condition, cells were switched to 18 mM galactose DMEM/F12 with B27 supplement, EGF, bFGF, 10 mg/l insulin, and 1% FBS for 6 h. Heavy membrane organelles were isolated, fixed in 4% PFA on ice for 20 min, and stained with Tom20-AF488 (Abcam, ab205486, 1:200), and V5-AF647 (Invitrogen 451098 1:200) diluted in Perm/Wash buffer (BD Biosciences, 554723). The heavy membrane fractions were resuspended in 1:5 homogenization buffer:PBS and analyzed on a Fortessa X-20. For DRP1 recruitment by single organelle analysis, BT25 control, NIK^−/−^, and DRP1^−/−^ cells were plated, metabolically switched to glucose or galactose for 3 or 6 h, stained for 20 min with 2.5 μM Mitosox^TM^ Red (Thermo Fisher Scientific, M36008) at 37 °C and fractionated as described above. The heavy membrane was then fixed with 4% PFA on ice for 20 min and then labeled with DRP1 (CST 8570S) conjugated to Alexa Fluor™ 488 (Invitrogen Zip AF488 Label Kit, Z11233). The DRP1-AF488/ Mitosox™ Red-labeled heavy membranes from the control, NIK^−/−^, and DRP1^−/−^ cells were then analyzed on the Fortessa X-20. DRP1 mitochondria coverage was determined using FlowJo Overton % Positive subtraction of DRP1-AF488^+^ signal between the control and NIK^−/−^ Mitosox™ Red^+^ organelles compared to the DRP1^−/−^ (negative control) signal. Single stained and unstained controls were used for all single organelle analysis experiments to determine spillover between channels. Mitochondria size measurements were established by calibrating the Fortessa with 1 μM, 2 μM, and 4 μM beads (Molecular Probes, F-13838, Oregon). Mitochondria analyses by flow cytometry were repeated in at least three independent experiments, each with ≥10,000 individual mitochondria events.

### Mouse xenograft assay

All animal experiments were done in compliance with IACUC, AAALAC and Texas A&M Health Science Center Biosafety guidelines using IACUC-approved Animal Use Protocols (# 2018-0483 and # 2015-0330). For orthotopic tumor inoculations, 0.5–1 × 10^6^ cells were injected into the right striatum of 4-6-week-old CD-1 nude mice. Mice were injected with luciferin and imaged for luminescence weekly post tumor inoculation on the IVIS Spectrum In Vivo Imaging System (Perkin Elmer). Tumor cells were either stably expressing luciferase or were labeled using a DiD Cytoplasmic Membrane dye (Biotium, abs/em = 644/665). For each cell type, ≥4 animals were injected.

### Histology and tumor imaging

Mice were deeply anesthetized for cardiac perfusion according to IACUC-approved protocols. Brains were dissected, fixed in 10% Neutral Buffered Formalin, sucrose cryoprotected, and sectioned on a cryostat (10 µm sections). H&E-stained sections were imaged on a VS120 Virtual Slide Microscope (Olympus).

### Protease protection assay

Protease protection assay was performed as previously described^52^. The heavy membrane fraction was mixed with 100 μl of ice-cold SEM buffer (10 mM MOPS/KOH [pH 7.2], 250 mM sucrose, 1 mM EDTA) at a concentration of 5 μg/μl. One aliquot of Swell-buffer-treated heavy membrane fraction and one aliquot of SEM-buffer-treated heavy membrane fraction were treated with 5.26 μl of Proteinase K (1 mg/ml in SEM buffer) and incubated on ice for 30 min. Protease activities were inhibited in all samples adding 5.26 μl of phenylmethylsulfonyl fluoride (PMSF) (200 mM in isopropanol) to each sample. Protein was precipitated from solution with 72% trichloroacetic acid (TCA) overnight on ice. The precipitate was collected by centrifugation at 28,000 × *g* for 30 min at 4 °C and washed two times with ice-cold PBS to remove any remaining supernatant and analyzed by immunoblot. For flow cytometry analysis, heavy membrane fractions were stained with 2.5 μM Mitosox^TM^ Red, and either left untreated or treated with Proteinase K, fixed for 20 min with 4% PFA, then stained with V5-AF647- (to detect NIK) and Tom20-AF488-conjugated antibodies before being analyzed with the Fortessa X-20. The heavy membrane fractions were first gated based on Mitosox Red^+^ organelles, then gated to detect Tom20-AF488-positive and V5-AF647-positive membranes.

### In vitro kinase assays

DRP1 GST constructs were a kind gift from the Kashatus lab^12^. GST-DRP1^518–736^ was expressed in *Escherichia coli* BL21 and purified using glutathione-sepharose-4B (GE Healthcare, #17075601). The GST fusion protein eluted from the beads via glutathione (Sigma-Aldrich, G4251) was dialyzed against PBS; 500 ng of GST-DRP1 was incubated with HA-NIK (NIK wild-type and NIK^K429A/K430A^)-bound beads in 25 μl of 1× Kinase Buffer (Cell Signaling Technology, 9802) and 20 μM ATP for 15 min at 37 °C. The kinase assay was terminated by the addition of LDS sample buffer containing a reducing agent for 7 min at 100 °C and resolved by SDS-Page. Additionally, ADP-Glo NIK kinase enzyme system (Promega, V4077) using purified recombinant human NIK protein (325aa-end) was used to perform kinase assays following the manufacturer’s instructions.

### 3D invasion assay

Invasion assays were performed as described previously^50,53^.

### Quantification and statistical analysis

Statistical analyses were carried out using GRAPHPAD PRISM software, and details can be found in the figure legends where applicable. The data presented here was considered statistically significant if *p* < 0.05. Tukey post-hoc tests were used for one-way and two-way ANOVA. For ANOVA, each group sample is from a normally distributed population, all samples are independent of each other and have a common variance.

## Supplementary information

Supplementary Figure Legends

Supplementary Fig. 1

Supplementary Fig. 2

Supplementary Fig. 3

Supplementary Fig. 4

Supplementary Fig. 5

Supplementary Fig. 6

Supplementary Fig. 7

Supplementary Fig. 8

## Data Availability

All data and materials generated in this study are available upon request.
