# Supplementary material for: NIK promotes metabolic adaptation of glioblastoma cells to bioenergetic stress
Source: Cell Death Dis. 2021 Mar 15;12(3):271. doi: 10.1038/s41419-020-03383-z (PMC7960998; doi:10.1038/s41419-020-03383-z)
Supplement: Supplementary file 1 — Supplementary Figure Legends [file 41419_2020_3383_MOESM1_ESM.docx]

**Supplementary Fig. 1.
Calculation of Mitochondria Size by flow, validation of GFP-NIK fusion protein function, and observed defects in OXPHOS in BT114 GBM cells. a,** Schematic of western blot protease protection assay (left) and single organelle flow cytometry (right). **b**, Representative western blot showing protease protection assay with indicated antibodies **c,** Left: Dot plot of calibration beads from 1-15 microns. Right: Standard curve used to calculate size of mitochondria run through the flow cytometer. **d,** Western blot analysis of cytosolic and nuclear fractions isolated from 293T cells transiently expressing CMV-GFP (empty vector control) or GFP-HA-NIK. Blots were probed with the indicated antibodies. Increased phosphorylated IKKα/β (p-IKKα/β) and p52 levels were observed in cells expressing GFP-HA-NIK, demonstrating functionality of the fusion GFP-HA-NIK construct. **e**, Representative Seahorse MitoStress plot showing the mean +/- SD values for BT114 control and NIK^-/-^ cells (n=3 replicates per cell line).

**Supplementary Fig. 2.**

**NIK is recruited to mitochondria fission sites in COS-7 cells
a,** Time-lapse images of COS-7 cells transiently expressing mito-BFP, mch-DRP1 and GFP-NIK were acquired at indicated time points. Grayscale images show mito-BFP
fluorescence with arrow indicating fission site. **b,** Line-scan analysis of mean fluorescence intensity shows highest GFP-NIK and mch-Drp1 localization at the mitochondrial fission site (lowest mito-BFP signal). **c-e**, Representative live cell confocal imaging performed on COS-7 cells transfected with Mito-BFP, GFP-NIK, and mCherry-DRP1 (mchDrp1). Cells were imaged for an hour after a media switch that was absent of glucose and contained 25mM galactose. **f-g**, Mander’s correlation coefficient (MOC) values for NIK (FITC) and mito-BFP (DAPI) (NIK:mitochondria), NIK (FITC) and DRP1(TRITC) (NIK:DRP1) and DRP1 (TRITC) and mito-BFP (DAPI) (DRP1:mitochondria) co-localization in the whole cell (**f**) and boxed ROI (**g**). Error bars indicate +/- SD of Mander’s coefficient taken over 3 consecutive frames in image analyzed. Two-way ANOVA followed by Tukey post-hoc test ** indicates p<.01, *** indicates p<.001, **** indicates p<.0001.

**Supplementary Fig. 3.**

**NIK forms a complex with Drp1.
a,** BT25 control, BT25 NIK^-/-^, BT25 NIK^-/-^ +NIK and BT25 NIK^-/-^  + NIK kinase-dead mutant K429A/K430A (NIK-KD) lysates were subjected to immunoprecipitation (IP) with control IgG or a Drp1 antibody, and then analyzed by immunoblotting with the indicated antibodies. **b,** 293T cells were transfected with HA-NIK wild-type (NIK) or NIK-KD (KD) expression vectors. Cell lysates were immunoprecipitated with control IgG or a Drp1 antibody, analyzed by immunoblotting with the indicated antibodies.

**Supplementary Fig. 4.**

**Characterization of BT25 IKKα/β^-/-^, IKKα/β^-/-^ NIK^-/-^, and Drp1^-/-^ cell lines.
a**, Representative Western blot of the IKKα/β^-/-^ cell lines generated by CRISPR/Cas9 editing. Clone 1 was used for this study. **b,** Immunoblot analysis of cytoplasmic and nuclear fractions from BT25 control and IKKα/β^-/-^ cells. BT25 IKKα/β^-/-^ cells demonstrate impaired nuclear translocation of p65, RelB, and p52 upon stimulation with Tweak and TNFα. **c,** Immunoblot analysis of whole cell lysates prepared from control and putative IKKα/β^-/-^ NIK^-/-^ cell lines. Clone 5 was used for this study. **d**, Drp1-/- cell lines. Clone 1 is shown in this study.

**Supplementary Fig. 5.**

**Drp1 phosphomimetics do not activate IKK/NF-κB
a,** Western blot with indicated antibodies characterizing constitutively active Drp1 mutant DRP1^S616E^ in a BT25 NIK^-/-^ CRISPR derived knockout (left). IKKα constitutively active mutant (lane 2), but not DRP1 constitutively active mutant (lane 3), activates p100 processing in BT25 NIK^-/-^ cell line (compare lanes 2 and 3 to lane 1). (Right) DRP1^S616A^ does not activate p100 processing compared to control BT25 cells. **b**, Representative cell viability data (propidium iodide exclusion) showing fold change cell death by shifting from 18 mM glucose to 18 mM galactose media for 48 hours (% dead cells in galactose/%dead cells glucose). n≥10,000 cells per condition at least n=3 replicates per condition. One-Way ANOVA followed by Tukey post-hoc analysis. a vs b p<.001, Data for Control and NIK^-/-^ taken from 5J.

**Supplementary Fig. 6.**

**Galactose Increases Basal Respiration, ATP Production and Spare Respiratory Capacity in GBM cells**Data graphed here is the same as in Figure 7, represented as fold change from each individual cell line’s basal respiration, ATP production, or spare respiratory capacity in glucose. **a-f,** Increases in basal respiration (a, d), spare respiratory capacity (b, e) and ATP production (c, f) are observed in GBM cell lines indicated above except for the basal respiration, ATP production, and spare respiratory capacity of BT25 DRP1^-/-^ cells and ATP production and spare respiratory capacity of BT25 NIK^-/-^ cells.. Paired t test, * indicates p<.05, ** indicates p<.01, *** indicates p<.001, **** indicates p<.0001.

**Supplementary Fig. 7.**

**Mitochondrial dynamics, OXPHOS genes, and the kinase function of NIK are all essential and contribute to GBM progression *in vivo***
**a,** Three-dimensional collagen invasion assay with indicated cell lines (mNIK indicates murine NIK), n=8 replicates. p<.0001 between all conditions a-e. Significance was determined by One-Way ANOVA followed by Tukey post hoc analysis. **b**, Heat map demonstrating genes essential for mitochondria dynamics, OXPHOS, glycolysis and hypoxia upregulated or downregulated at the tumor leading edge compared to the tumor core of 8 human GBM tumors. Data taken from IVY GAP database.

**Supplementary Fig. 8.**

**NIK depletion rewires GBM cells toward glycolysis independent of IKK.
a-b,** ECAR measurements in BT25 cell lines for Seahorse glycolysis stress. Glycolysis is measured as the difference in basal ECAR in glucose free media from the peak ECAR immediately following 5 mM glucose injection. **c,** ECAR measurements in BT1114 cell lines for Seahorse glycolysis stress test. **d-e**, Representative graphs of glycolysis measurements for BT25 Control, DRP1^-/-^, NIK^-/-^, NIK^-/-^ DRP1^616E^ (**d**) and BT25 Control, IKKα/β^-/-^, IKKα/β^-/-^, NIK^-/-^ cell lines (**e**). One-way ANOVA followed by Tukey-post hoc analysis. Different letters indicate statistical significance. a vs b p<.0001, a vs c p<.001, b vs c, p<.0001. **f,** Representative graph of glycolysis measurements for BT114 control and NIK^-/-^ GBM cell lines as measured by the Seahorse glycolysis stress test. Student t-test, **** indicates p<.0001.
