# Supplementary figures and images for: NIK promotes metabolic adaptation of glioblastoma cells to bioenergetic stress
Source: Cell Death Dis. 2021 Mar 15;12(3):271. doi: 10.1038/s41419-020-03383-z (PMC7960998; doi:10.1038/s41419-020-03383-z)

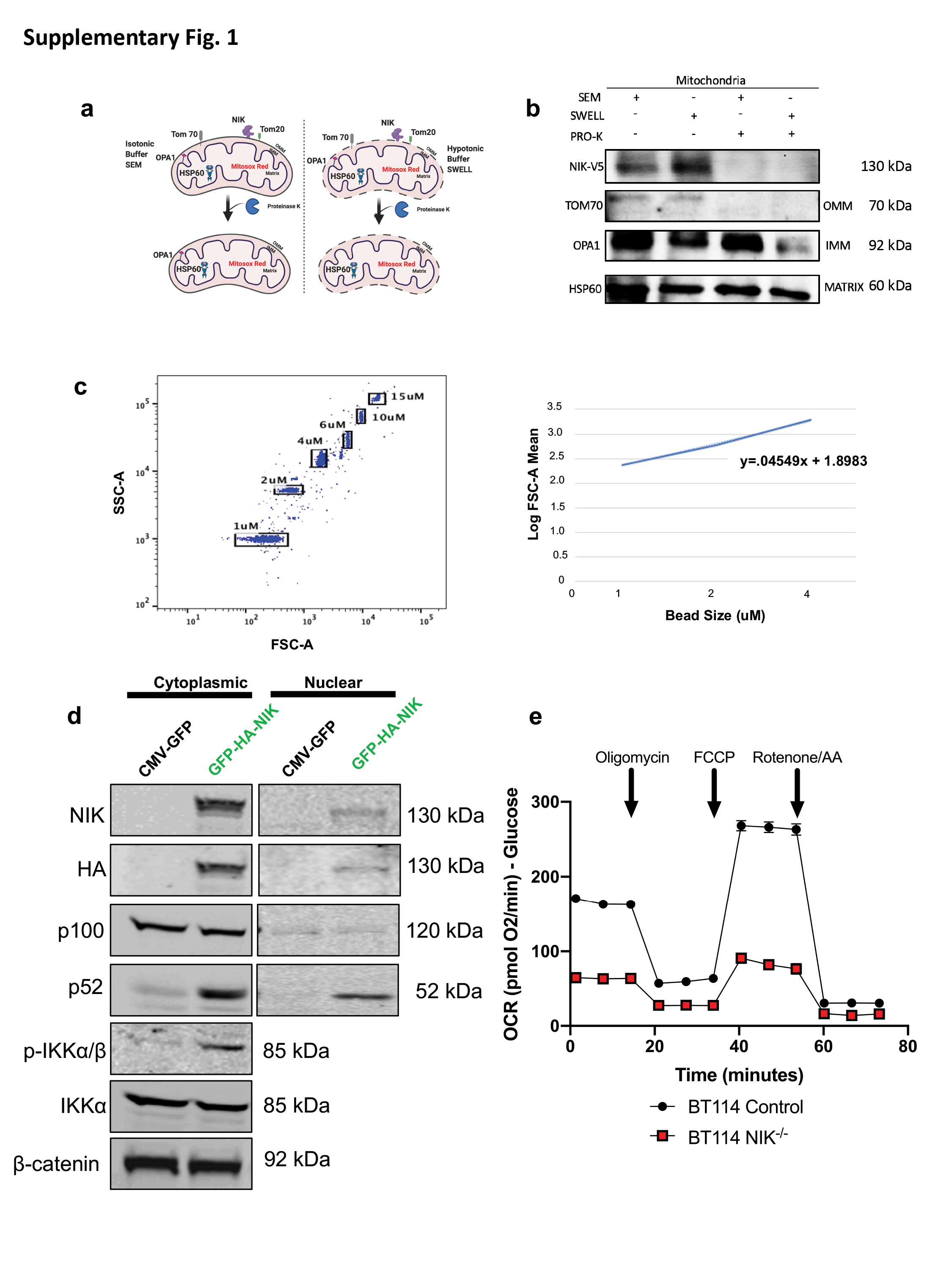

Supplement: Supplementary file 2 — Supplementary Fig. 1 [file 41419_2020_3383_MOESM2_ESM.png]

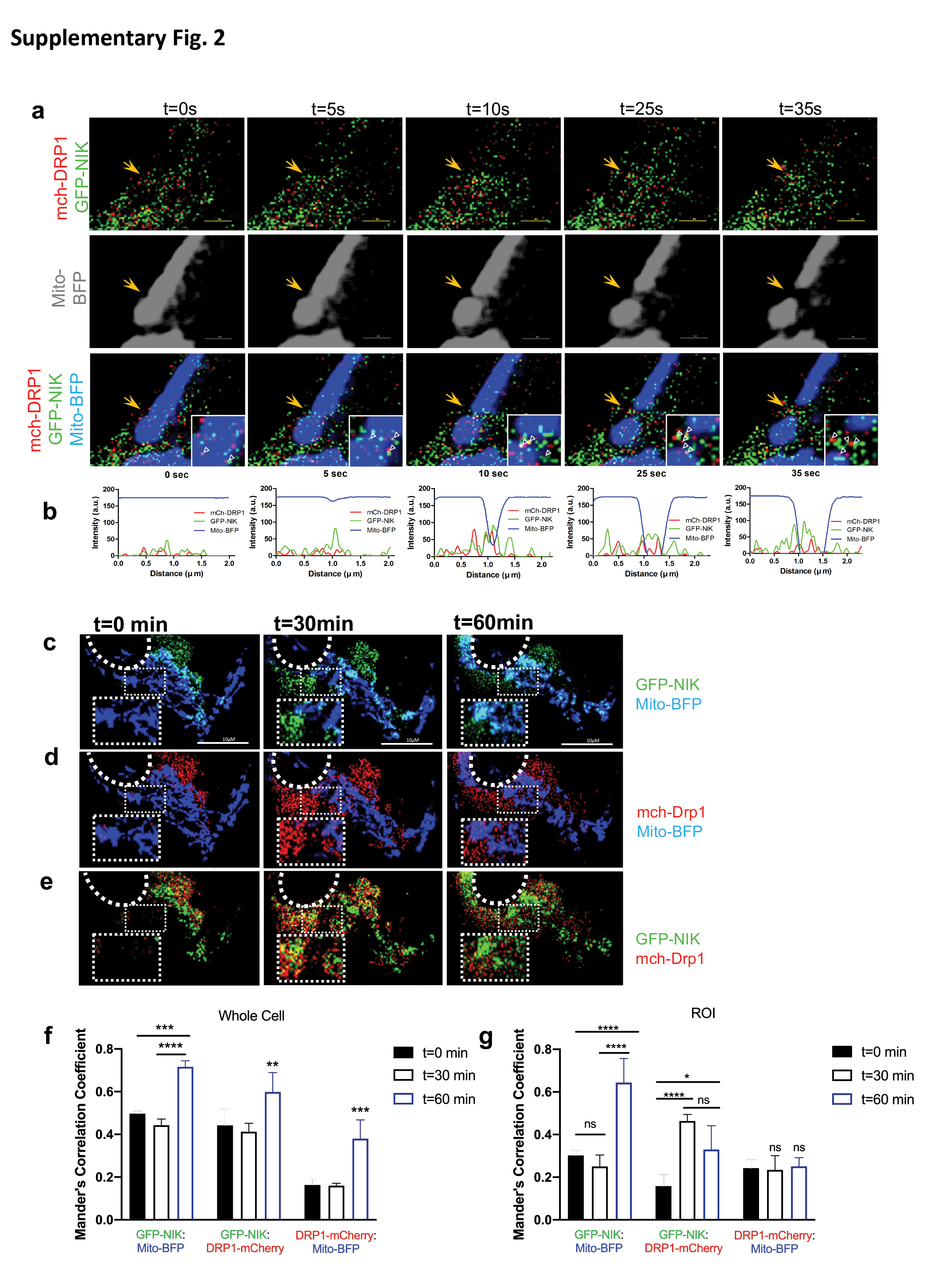

Supplement: Supplementary file 3 — Supplementary Fig. 2 [file 41419_2020_3383_MOESM3_ESM.png]

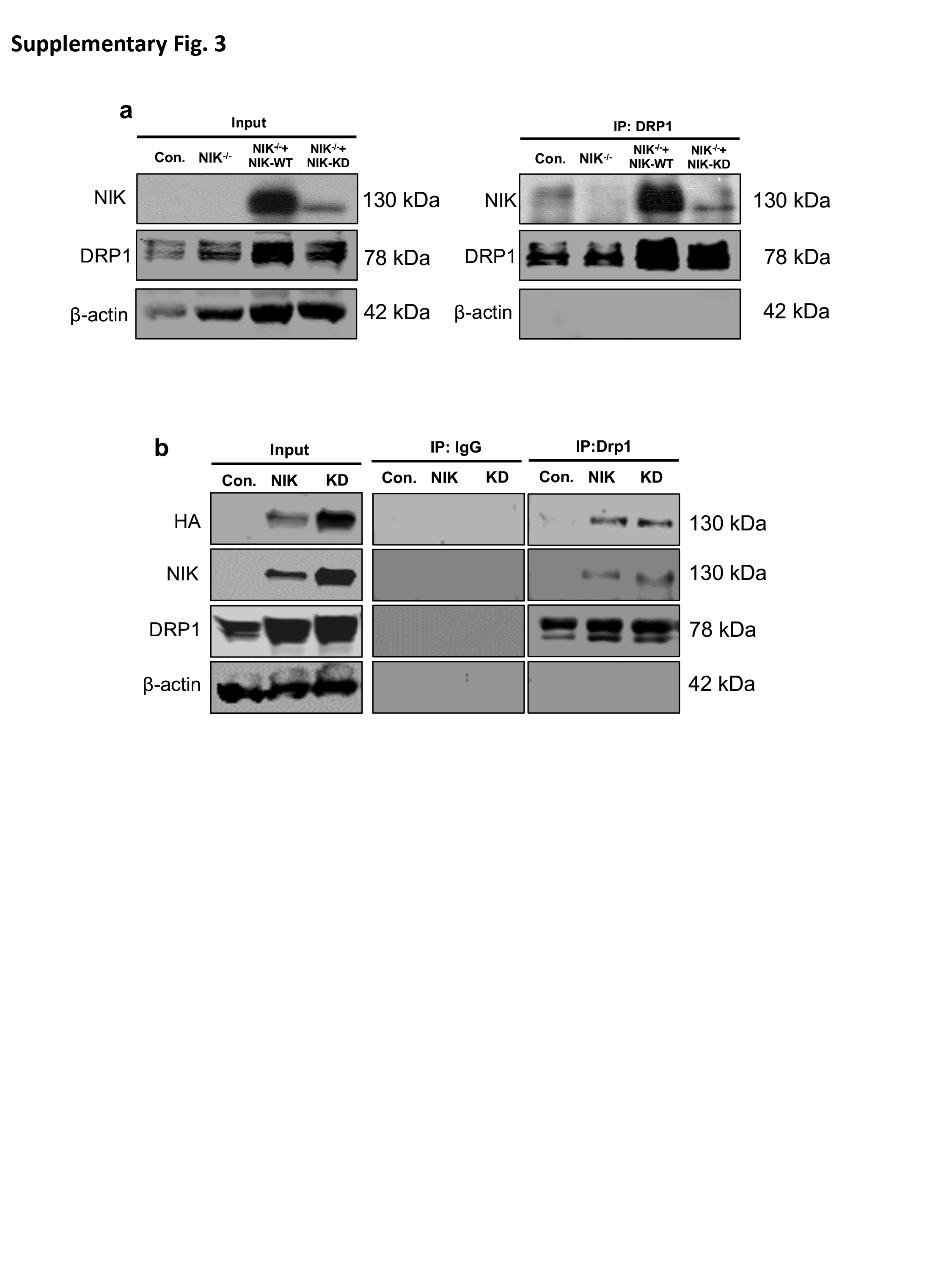

Supplement: Supplementary file 4 — Supplementary Fig. 3 [file 41419_2020_3383_MOESM4_ESM.png]

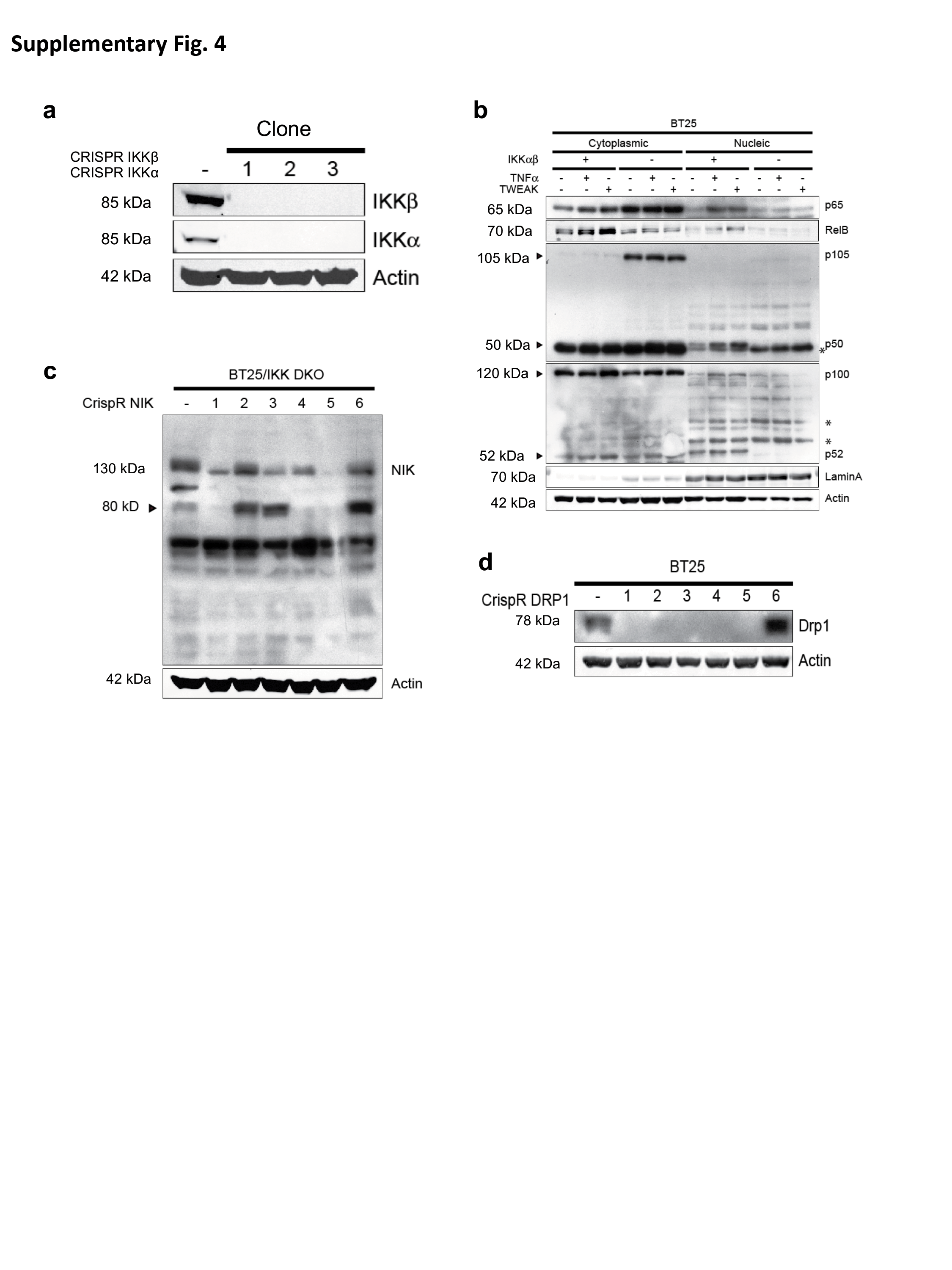

Supplement: Supplementary file 5 — Supplementary Fig. 4 [file 41419_2020_3383_MOESM5_ESM.png]

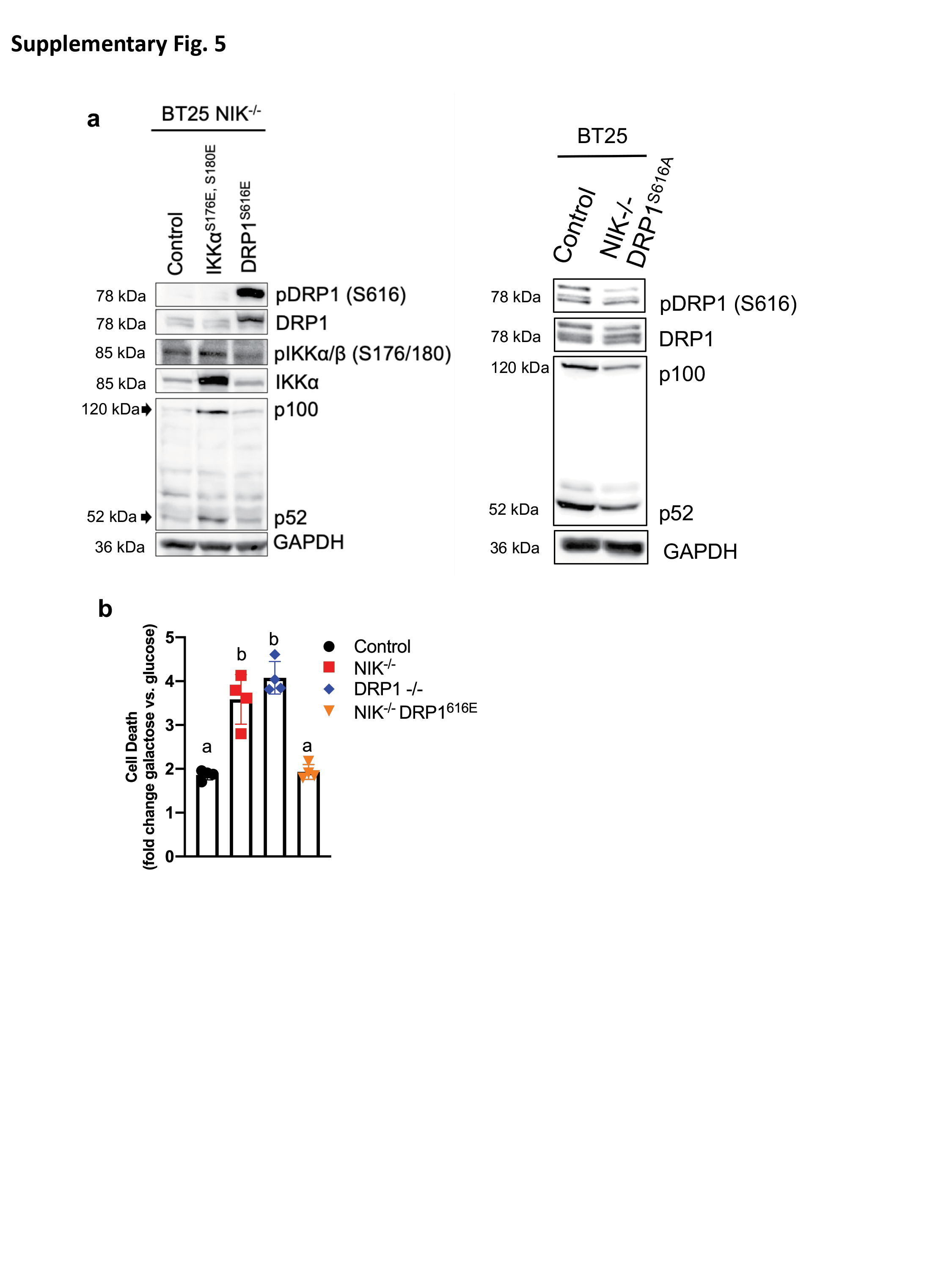

Supplement: Supplementary file 6 — Supplementary Fig. 5 [file 41419_2020_3383_MOESM6_ESM.png]

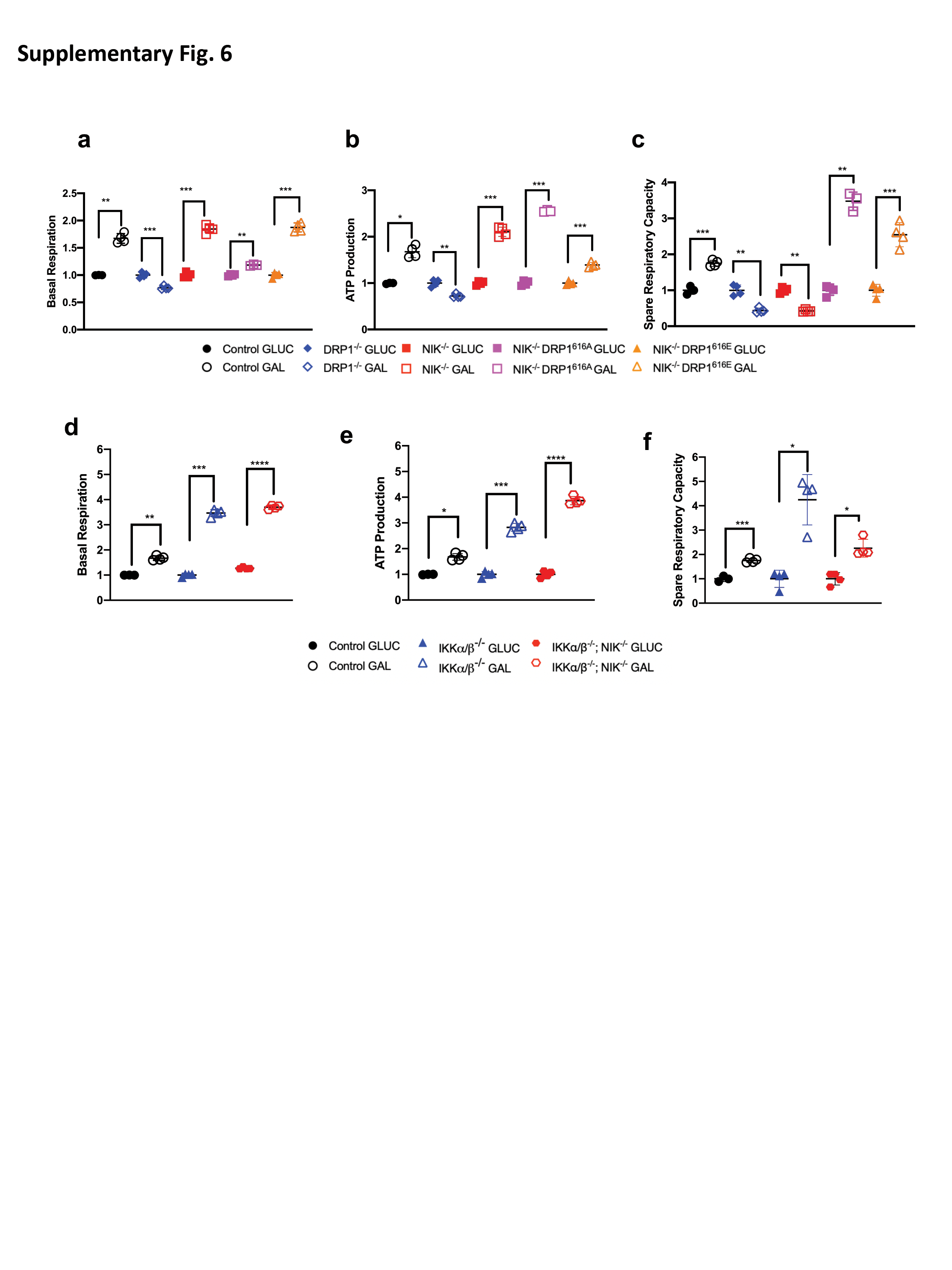

Supplement: Supplementary file 7 — Supplementary Fig. 6 [file 41419_2020_3383_MOESM7_ESM.png]

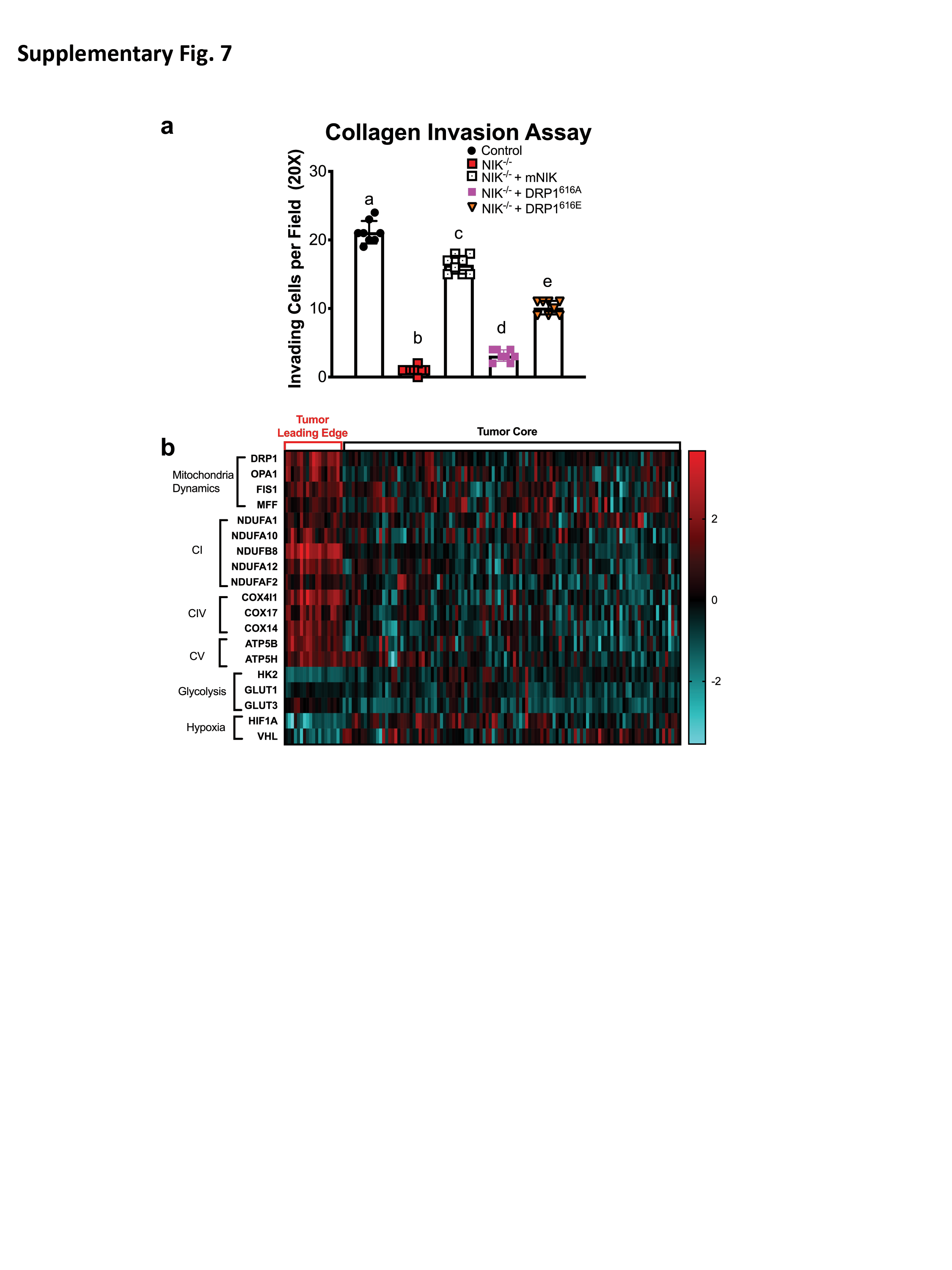

Supplement: Supplementary file 8 — Supplementary Fig. 7 [file 41419_2020_3383_MOESM8_ESM.png]

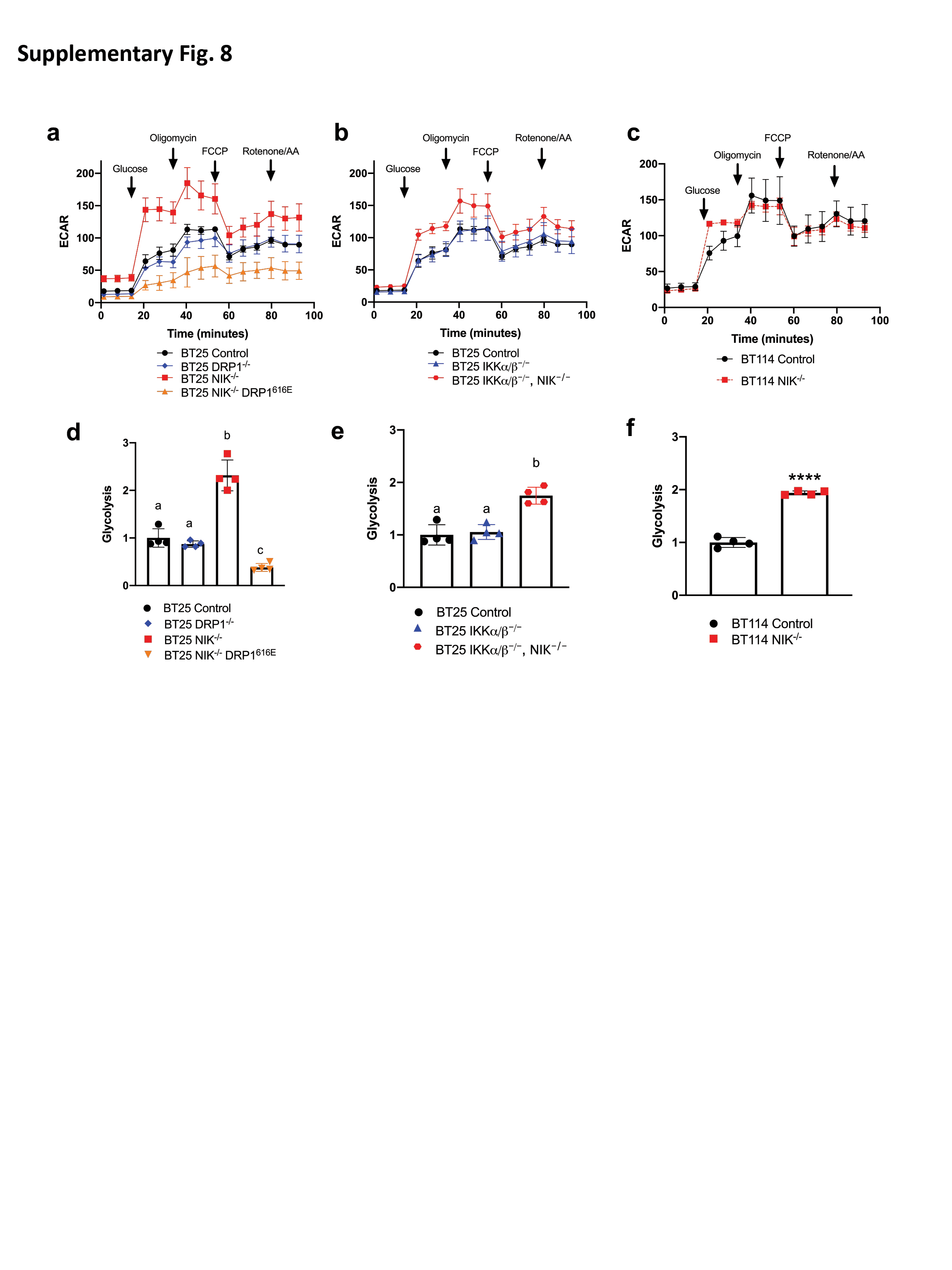

Supplement: Supplementary file 9 — Supplementary Fig. 8 [file 41419_2020_3383_MOESM9_ESM.png]
